# Biased Inter-columnar Communication and Short-Term Plasticity in Mouse Barrel Cortex

**DOI:** 10.1523/ENEURO.0109-26.2026

**Published:** 2026-09-11

**Authors:** John M. Judge, Meyer B. Jackson

**Affiliations:** ^1^Biophysics PhD Program, University of Wisconsin-Madison, Wisconsin Institutes for Medical Research, Madison, Wisconsin 53705; ^2^Department of Neuroscience, University of Wisconsin-Madison, Wisconsin Institutes for Medical Research, Madison, Wisconsin 53705

**Keywords:** genetic targeting, sensory filtering, somatosensory cortex, voltage imaging, whiskers

## Abstract

Each barrel of the barrel cortex (BC) receives thalamic input primarily from a single whisker. Barrels then communicate through intracortical circuitry to integrate input from multiple whiskers and perform processing functions such as spatial filtering, motion sensing, and object localization. To investigate the circuits that enable barrels to communicate independently of extracortical and inter-areal influences, we prepared slices of somatosensory cortex from mice of both sexes with a hybrid voltage sensor (hVOS) targeted to Scnn1a excitatory neurons in cortical layer 4 (L4). We then imaged voltage responses of this population of neurons to electrical stimulation. Imaging tracked activity initiated in L4 of one barrel spreading into layer 2/3 (L2/3) and then to L4 of neighboring barrels. AMPA receptor blockade eliminated this spread, suggesting that L4 signaling to neighboring barrels employs an L4→L2/3→L4 excitatory synaptic relay. Blocking AMPA receptors also enhanced some responses, revealing intra- and inter-barrel feedforward inhibition. Both coronal and sagittal slices presented the layout of barrels, which aligned with facial whisker organization. We assessed inter-barrel communication in different directions and found it to be isotropic in response amplitude, half-width, and conduction velocity. However, latency was longest for communication to caudal barrels. Furthermore, paired-pulse depression was strongest and recovery slowest for inter-barrel communication related to exploratory protraction. Such biases have the potential to contribute to direction-sensitive processing. Anisotropy in short-term plasticity can tune BC microcircuits to preserve temporal fidelity and filter selectively according to direction.

## Graphical abstract

**Figure:**
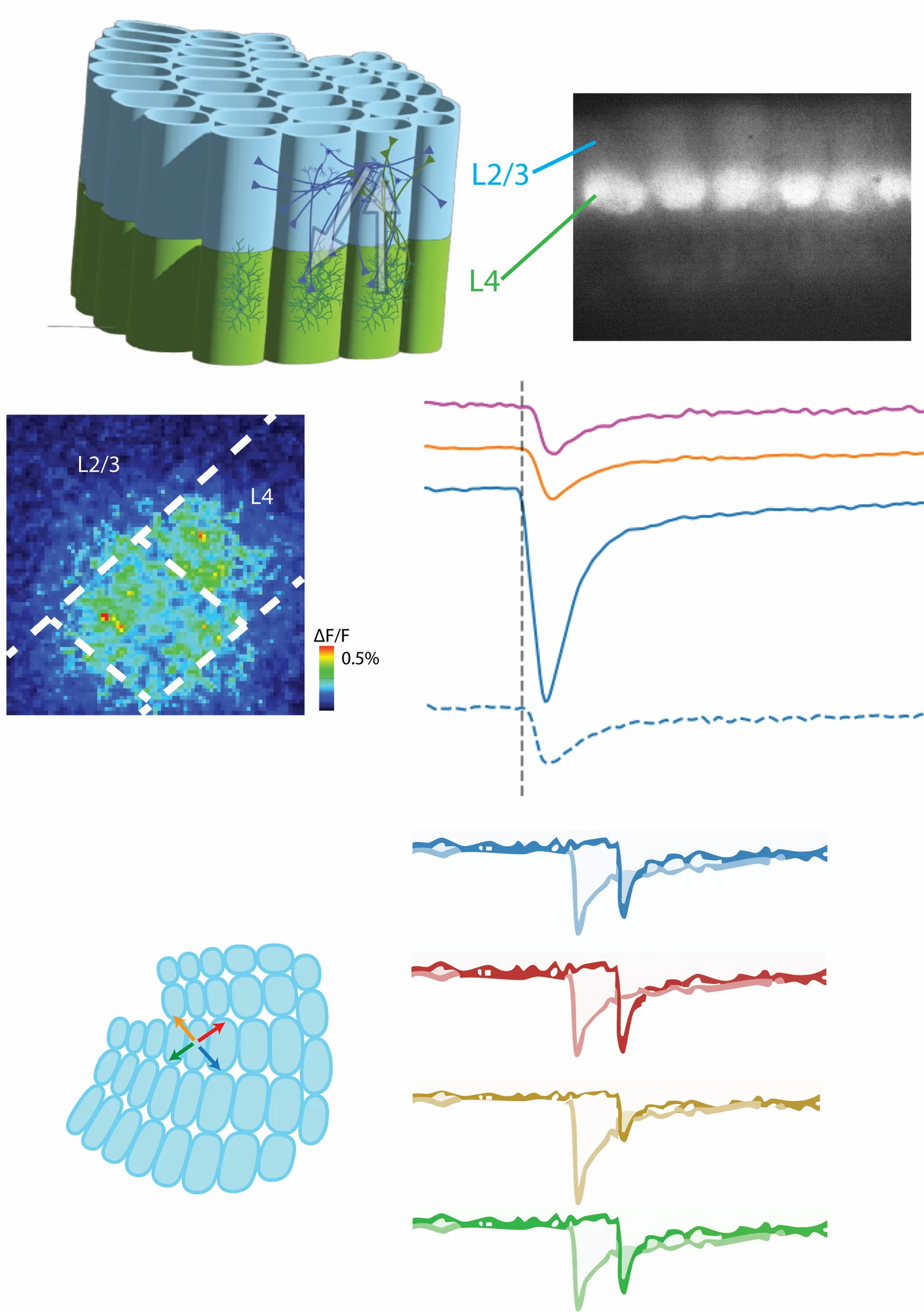
Visual Abstract

## Significance Statement

Sensory processing of spatiotemporal whisking patterns by the barrel cortex depends on complex interactions within the cortex and with extracortical areas. How intracortical circuits contribute to this processing is not well understood. Cell type-specific voltage imaging in anatomically aligned slices of somatosensory cortex enabled the investigation of how barrels communicate. We identified a layer-specific synaptic circuit that mediates communication between barrels. Response latency and short-term synaptic depression of this inter-barrel relay vary with propagation direction. This direction dependence suggests that intracortical communication is tailored to the processing of natural whisker motion. By linking synaptic properties and circuit connectivity between barrels to anatomically aligned whisker architecture, this work advances our understanding of how neocortical networks process sensory input.

## Introduction

The somatotopic map of mystacial whiskers in rodent primary somatosensory cortex provides a model for modular cortical processing (Woolsey, 1970; Egger et al., 2012; Stüttgen and Schwarz, 2018). Each whisker maps primarily to one barrel within the barrel cortex (BC), following the canonical thalamocortical path (Lübke et al., 2000; Viaene et al., 2011). How communication between barrels contributes to sensory processing remains poorly understood. Previous work has examined the in vivo flow of sensory information between layers and barrels (Armstrong-James et al., 1992; Zhang and Alloway, 2005), uncovering functional and morphological asymmetries between rows and arcs (Adesnik and Scanziani, 2010; Feldmeyer, 2012; Staiger and Petersen, 2021). However, corticothalamic feedback makes it difficult to isolate thalamocortical and cortical contributions to multiwhisker integration, particularly in L4 (Simons, 1978; Fox et al., 2003; Kwegyir-Afful et al., 2005; Sun et al., 2006; Lam and Sherman, 2009; Hill and Greenfield, 2014; Patel, 2019).

Behavioral studies implicate multiple circuit loci in the processing of sensory input. Directional bias in sensory input may require intracortical synapses to implement temporal filtering (Fortune and Rose, 2001), in order to operate on thalamocortical-transformed input at exploratory whisking frequencies of 1–5 Hz and small-angle foveal whisking frequencies of 15–25 Hz (Adibi, 2019). Furthermore, these computations vary across the somatotopic map, reflecting barrel specialization (Woolsey, 1970; Hobbs et al., 2016). In vivo studies show spatial or directional preferences to whisker deflections (Vilarchao et al., 2018; Hubatz et al., 2020), as well as phase-dependent biases during active whisking (McCasland et al., 1991; Bermejo et al., 2002; Leiser and Moxon, 2007; Curtis and Kleinfeld, 2009; de Kock and Sakmann, 2009; Kleinfeld et al., 2016). While little is known about how inter-barrel microcircuits can contribute to these anisotropies, recent progress has indicated that synaptic depression variations within the barrel microcircuit can implement spatial biases in filtering (Argunşah et al., 2026).

Experiments in brain slices can reveal intracortical communication in isolation from sensory input and have shown that electrical stimulation in L4 evokes activity that propagates to supragranular layers (Petersen and Sakmann, 2001; Laaris and Keller, 2002; Sato et al., 2008; Hill and Greenfield, 2014), with little if any spread to L4 of adjacent barrels except when inhibition is blocked (Sato et al., 2008). Here we used this approach to investigate a subpopulation of neurons in these microcircuits and to ask how activity spreads between barrels. We targeted a hybrid voltage sensor (hVOS) to L4 excitatory neurons (spiny stellate and pyramidal) in mouse BC and prepared cortical slices to image the membrane potential changes of neurons that receive the primary thalamic input. Stimulation in L4 activated multiple barrels in L4. Excitatory synapse blockade isolated barrels and revealed that communication between barrels depends on inter-barrel synaptic transmission. We then explored the possibility that inter-barrel communication can be biased in a manner relevant to sensory processing. We investigated response characteristics, velocities, and paired-pulse synaptic depression in BC microcircuits and evaluated inter-barrel connections in different directions to gain insight into isotropic and anisotropic aspects of inter-barrel communication.

## Materials and Methods

### Animals

Ai35-hVOS1.5 [C57BL/6-Gt(ROSA) 26Sortm1 (CAG-hVOS1.5)Mbja/J; JAX #031102] Cre reporter mice were bred with Scnn1a-tg3-Cre driver mice [B6;C3-Tg(Scnn1a-cre)3Aibs/J; JAX # 009613] to create Scnn1a::hVOS mice with hVOS probe expression (Chanda et al., 2005; Bayguinov et al., 2017) in L4 excitatory neurons (Madisen et al., 2009; Scala et al., 2019). All animal procedures were performed in accordance with the University of Wisconsin animal care committee's regulations (protocol: M005952).

### Slice preparation

Male and female mice were deeply anesthetized with isoflurane and killed via cervical dislocation. Dissected brains were placed into ice-cold cutting solution (in mM: 10 glucose, 125 NaCl, 4 KCl, 1.25 NaH_2_PO_4_, 26 NaHCO_3_, 6 MgSO_4_, 1 CaCl_2_) bubbled with 95% O_2_/5% CO_2_. We cut either ∼6 coronal or ∼4 sagittal 200 μm slices per brain hemisphere, with a Leica VT1200S Vibratome and placed them into 95% O_2_/5% CO_2_-bubbled artificial cerebrospinal fluid (ACSF, same as cutting solution except 1.3 mM MgSO_4_, 2.5 mM CaCl_2_, 4 μM DPA) for at least 1 h (Scheuer et al., 2023).

### Voltage imaging

Slices continuously perfused with ACSF at room temperature were viewed using an Olympus BX51 microscope and XLUMPlanFl 20× objective (NA 1.0). Slices were illuminated by an LED (435 nm peak emission; Prizmatix) and images acquired with a DaVinci 2K CMOS camera (2 kHz, 512 × 160 pixels binned/cropped to 80 × 80; RedShirt Imaging). Gradient contrast images were acquired with a high-resolution Kiralux CMOS camera (Thorlabs), and a movable mirror in a dual-port adapter was used to switch between the cameras. Image acquisition was controlled by Turbo-SM software (RedShirt Imaging/SciMeasure) and a C++ application for data processing, PhotoZ (Chang, 2006). A Python application (OrchestraZ) automated the workflow. Displayed traces of fluorescence versus time were averages of five trials, at 15 s intervals.

### Electrical stimulation

A triggered pulse train generator (Prizmatix) drove a stimulus isolator (World Precision Instruments) to deliver pulses (50–250 μA, 200 μs) through a fire-polished ACSF-filled KG-33 glass microelectrodes (King Precision Glass) with tip diameters ∼6–8 μm. Electrodes were positioned visually in the indicated locations with a micromanipulator.

### Analysis and statistics

We analyzed data from 2–3 barrels per slice and 1–4 slices per animal using 11 males (21 slices, 48 barrels) and 11 females (23 slices, 56 barrels); mouse age ranged from 4 to 13 weeks with an average of 7.8 ± 1.7 weeks. The contribution of animals to each experiment was as follows: 4 animals for L2/3 stimulation with NBQX in coronal slices; 7 animals for L4 stimulation with NBQX in coronal slices; 6 animals for PPR in coronal slices; 5 animals for PPR in sagittal slices; 3 animals for L2/3 and L4 stimulation with NBQX in sagittal slices; and 3 animals for varying stimulation current. The total was 28 animals, as 6 contributed slices to two different experiments.

The data acquisition program PhotoZ (Chang, 2006) measures peak Δ*F*/*F* and implements baseline subtraction, temporal and spatial filtering, and linearly interpolated measurements of latency and half-width. The latency is the time from the stimulation pulse to the half-peak amplitude. Time windows for analysis were from 3 ms before stimulation to 50 ms poststimulation, with independent samples obtained as barrel-averaged measurements. Regions with lengths of at least 50 μm showing constant velocity were identified for linear regression and velocity measurement. Velocity was calculated with the SciPy.stats Python library with linear regression on latency versus distance along L4, using points from seven or more regions of interest (ROIs) in each barrel. Pixels within 50 μm of the stimulation site were excluded. Septa between barrels were also excluded due to their dim fluorescence.

We tested multiple groups for significance with one-way or two-way ANOVA (SciPy.stats). If ANOVA indicated significant variation, Shapiro–Wilk tests confirmed normality before we used Tukey's honestly significance difference (HSD) test for pairwise comparisons (Statsmodels, Python). We tested the variance attributed to random effects with a hierarchical mixed-effect linear regression model implemented in R [response metric ∼ fixed effects + (1 | Litter) + (1 | Litter:Animal) + (1 | Litter:Animal:Slice)]. Litter variances (Δ*F*/*F*: $2.5\times 10^{-20}$, latency: $3.0\times 10^{-8}$, half-width: 2.69), animal variances (Δ*F*/*F*: $7.2\times 10^{-7}$, latency: $7.3\times 10^{-1}$ half-width: 0.641), and slice-to-slice variances (Δ*F*/*F*: $1.2\times 10^{-6}$, latency: $1.4\times 10^{-1}$, half-width: 2.66) accounted for much less than residual variance (Δ*F*/*F*: $7.8\times 10^{-7}$ latency: 6.56, half-width: 88.2), indicating no significant pseudoreplication effects between slices of the same animal or litter (Lazic, 2010). In half-width and latency, variances had negligible sensitivity to all random effects, but Δ*F*/*F* had a slice variance an order of magnitude greater than the other two random effects combined. Nonetheless, averaging at the barrel level ruled out any possible pseudoreplication effects from slice variance, with low chance of pseudoreplication from litter or animal except for a moderate chance of clustering of half-width due to animal variance (animal half-width variance percentage: 11%; all others: 0–3%). However, the present study did not find any significant dependence of half-width on any factor. Because animal-to-animal variation includes age-based variation, this also eliminated age as a potential predictor.

A comparison of male versus female mice showed no significant differences in latency, half-width, velocity (Welch's *t* test, *T* < 0.001, *p* = 1.0 for all), or paired-pulse ratio (PPR) (multivariate linear regression, *F* = 0.55, *p* = 0.46). Mice aged 30–60 d showed no significant correlation between these metrics and age: response latency, half-width (linear regression, L2/3: latency: *p* = 0.23, half-width: *p* = 0.48; L4: latency: *p* = 0.16, half-width: *p* = 0.57), velocity (linear regression, *p* = 0.66), or PPR (multivariate linear regression, *F* = 0.006, *p* = 1.0). In cases where only two groups were compared, we used one-sided Welch's *t* tests (SciPy.stats). We also used one-sample one-sided *t* tests (SciPy.stats) to compare the mean against null hypothesis values where relevant.

We designed our sample sizes to detect differences on the order of at least 15%. To achieve a standard power level (0.8) and false-positive rate of 5%, allowing for an uncertainty of 8%, we calculated that a minimum sample size of 8 is needed: $n_{2}=n_{1}=\;\frac{\sigma_{1}^{2}+\sigma_{2}^{2}/K}{\Delta^{2}}(z_{1-\alpha /2}+z_{1-\beta /2}{)}^{2}=4$. Table 1 reports effect sizes and confidence intervals; *p* values, *f* statistics, and *t* statistics are given in the Results.

**Table 1.** Statistics table

Data structure	Statistical tests	Effect size (*ηp*^2^)	Confidence intervals
Δ*F*/*F* ratio differences; normal distributions	1-way ANOVA, Tukey's HSD	0.04	C vs D [−0.32 0.28]
			C vs R [−0.38 0.22]
			C vs V [−0.24 0.33]
			D vs R [−0.38 0.26]
			D vs V [−0.24 0.37]
			R vs V [−0.17 0.43]
Half-width differences; normal distributions	1-way ANOVA, Tukey's HSD	0.03	C vs D [−5.8 4.8]
			C vs R [−6.4 4.2]
			C vs V [−4.1 5.9]
			D vs R [−6.1 5.1]
			D vs V [−3.9 6.7]
			R vs V [−3.3 7.2]
Velocity differences; normal distributions of linear regression slopes	1-way ANOVA, Tukey's HSD	0.05	C vs D [−141 63]
			C vs R [−87 140]
			C vs V [−78 55]
			D vs R [−64 196]
			D vs V [−64 120]
			R vs V [−142 67]
Half-amplitude latency differences; normal distributions	1-way ANOVA, Tukey's HSD	0.48	C vs D [10.6 38.4]
			C vs R [−14.4 13.5]
			C vs V [−1.7 24.6]
			D vs R [−39.6 −10.2]
			D vs V [−27.0 0.88]
			R vs V [−2.1 25.79]
Paired-pulse ratio (PPR); normal distributions of exponential fit PPR intercept parameter at each IPI	2-way ANOVA, Tukey's HSD	0.46	C vs D [−0.37 −0.12]
			C vs R [−0.18 0.05]
			C vs V [−0.19 0.06]
			D vs R [0.06 0.30]
			D vs V [0.05 0.31]
			R vs V [−0.11 0.12]
Paired-pulse ratio (PPR); normal distribution of exponential fit time constant parameter at each IPI	2-way ANOVA, Tukey's HSD	0.41	D vs R [−358 96]
			D vs V [−325 161]
			R vs V [−169 267]

C, caudal; D, dorsal; R, rostral; V, ventral. *p* values, *T* statistics, and *F* statistics are given in the Results. Detailed information about significance tests is given in Materials and Methods.

## Results

Barrel organization in coronal and sagittal slices is highlighted by Scnn1a labeling, which has been shown to target ∼50% of the excitatory neurons in mouse L4 with a Cre reporter closely related to that used here (Wang et al., 2024). This distinctive pattern of labeling enabled us to align barrels and whiskers. We used voltage imaging to follow the spread of excitation within and between barrels, evaluating the contributions of excitatory synaptic transmission to inter-barrel communication along an L4→L2/3→L4 relay by blocking excitatory synapses. We then determined response parameters for inter-barrel communication in different directions. Finally, we assessed short-term plasticity between barrels along behaviorally relevant axes.

### Barrel visualization and alignment

**Figure 1.**
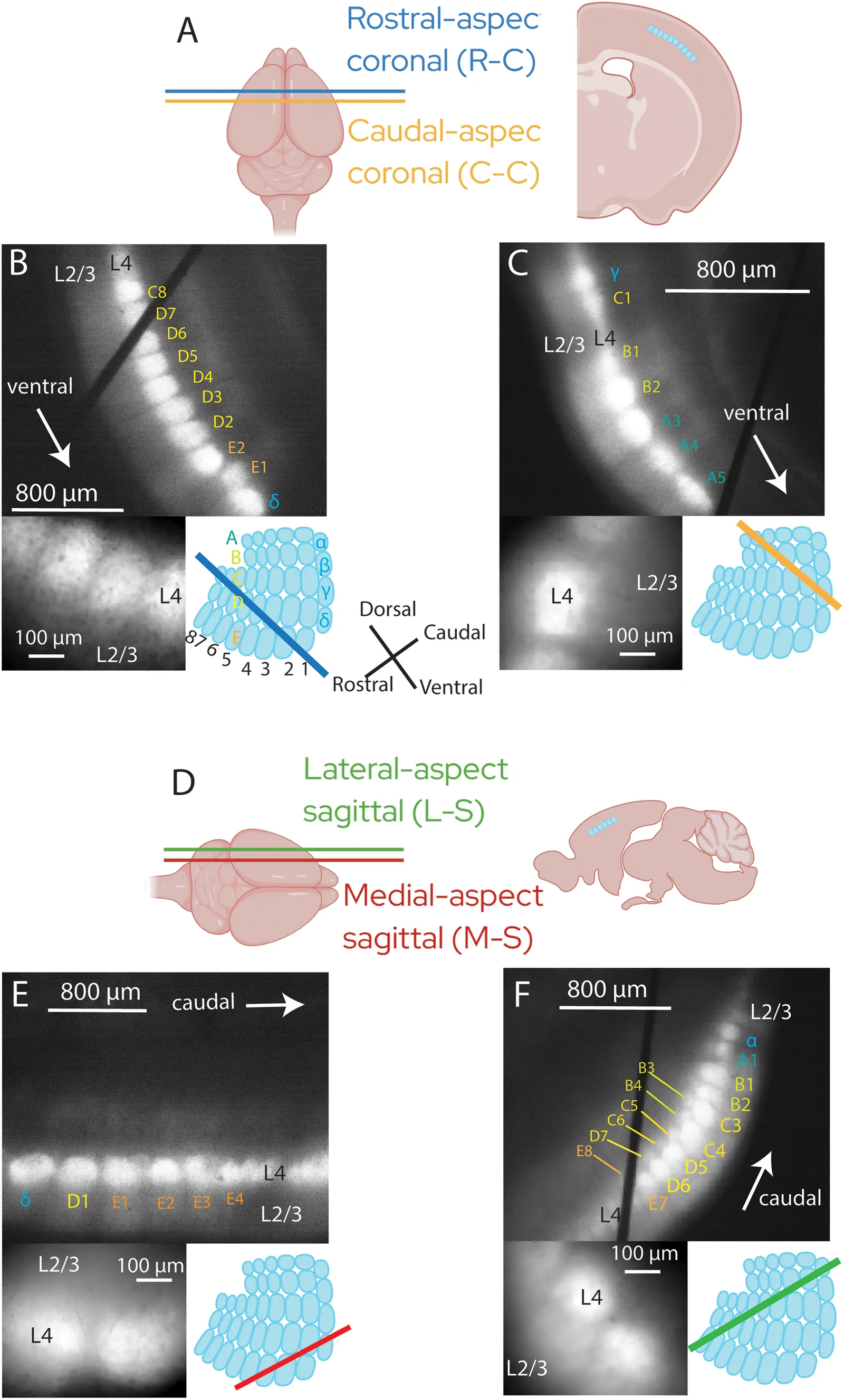
Anatomical orientation and slicing strategy. ***A***, Left, Dorsal view of mouse brain in the coronal plane: rostral-aspect coronal (R-C, blue) and caudal-aspect coronal (C-C, orange) sections. Right, Coronal slice with barrels highlighted as blue (created with BioRender). ***B***, ***C***, Top, Low-magnification fluorescence (arising from hVOS probe in Scnn1a-expressing neurons) from R-C (***B***) and C-C (***C***) slices, with annotations for barrel, barrel relationship, and orientation. Bottom left, Higher magnification of the corresponding slice. Bottom right, Diagram of tangential section of barrel cortex; blue and orange lines indicate approximate location of respective slicing planes for R-C and C-C in the barrel field. ***D***, Left, Dorsal view of mouse brain showing slicing planes: lateral-aspect sagittal (L-S, green) and medial-aspect sagittal (M-S, red). Right, Illustration of barrels in a sagittal slice (created with BioRender). ***E***, M-S slices. ***F***, L-S slices, paralleling panels and annotations in ***B*** and ***C***. Barrel row and arc assignments are given to within one row or arc; this uncertainty was acceptable because determining only aspect and plane classification is needed. Note that the black fibers visible in the low magnification images are threads of the harps used to anchor slices. See Extended Data Figure 1-1 for analysis barrel dimensions.

The BC columnar organization is visible in low magnification fluorescence images of somatosensory cortex slices from Scnn1a::hVOS mice (Fig. 1). Fluorescence from hVOS probe is concentrated in L4 barrel hollows. Our choice of 200 µm for slice thickness limited fluorescence from barrels outside of the microscope focal plane and matched the sum of our measurements of barrel width plus septal width (154–200 µm; barrel dimensions are illustrated in Extended Data Fig. 1-1*A* and plotted in Extended Data Fig. 1-1*B*–*E*).

10.1523/ENEURO.0109-26.2026.f1-1 figure 1-1 **Barrel dimensions across slice types**. **A.** Bright field (left) and resting fluorescence (right) images featuring barrel width (light blue) and height (dark blue), and septum width (yellow). The L2/3-L4 boundary is determined based on the presence of septal columns and higher fluorescence in L4 than in L2/3. **B**. Comparison of septal width among R-C, C-C, and M-S slices (note that L-S slices were not included, see text). **C**. Plot of barrel width versus septum width with line from linear regression (p < 0.05). **D-E.** Barrel height and width differ across slice types (M-S barrel height is greater than that of R-C and C-C; R-C barrels were narrower than M-S barrels). Each point indicates one barrel or septum; lines show mean ± SEM. Significance assessed by Welch’s t-test. Download figure 1-1, TIF file.

We initially used the BrainGlobe atlas of mouse BC (Claudi et al., 2020) to evaluate directionality of inter-barrel communication revealed by voltage imaging, sampling the atlas in 12 simulated coronal or sagittal sections to estimate the probability *P(row)* of two adjacent barrels being in the same row, *P(arc)* of being in the same arc, and *P(diagonal)* of being in neither the same row nor the same arc. We obtained *P(row)* = 0.90 in R-C slices, 0.50 in C-C slices, and 0.58 in M-S slices, while *P(arc)* = 0.06, 0.40, 0.17 (note *P(row)* + *P(arc)* + *P(diagonal)* = 1). The lack of a clear alignment along row or arc in these slice planes is in part due to the curvature of the barrel field.

Coronal slices preserved dorsal-ventral connections (Fig. 1*A–C*) and sagittal slices preserved rostral-caudal connections (Fig. 1*D–F*). However, as we confirmed with the atlas, these slices do not strictly preserve rows and arcs due to the curvature of the barrel field grid and its rotation from the whisker grid. This is consistent with typical tilt used to cut tangential BC slices (Sumser et al., 2017). Accounting for this alignment, we categorized our slices based on aspect and orientation. Figure 1, *B*, *C*, *E*, and *F*, shows barrel relationships in coronal and sagittal slices, respectively, accompanied by higher magnification images (bottom left) and tangential diagrams of BC with slice planes drawn (bottom right). We divided slices into four groups: rostral-aspect coronal (R-C slice; Fig. 1*A*,*B*), caudal-aspect coronal (C-C slice; Fig. 1*A*,*C*), lateral-aspect sagittal (L-S slice; Fig. 1*D*,*E*), and medial-aspect sagittal (M-S slice; Fig. 1*D*,*F*). R-C slices tend to present a cross section of rows C, D, or E (labeled in Fig. 1*B*, bottom right), which curve into the rostrodorsal direction in the rostral aspect (Fig. 1*A*, right). These rows have narrow septa and a higher barrel count (7–10 per slice). In contrast, C-C slices present fewer, larger barrels and septa (Fig. 1*C*). Sagittal slices originated from either aspect (Fig. 1*E*, M-S; Fig. 1*F*, L-S), with lateral slices containing barrels corresponding to ventral whiskers and medial slices containing barrels corresponding to dorsal whiskers (Petersen et al., 2020). In L-S slices the slice plane becomes nearly tangential, making their organization ambiguous. For this reason L-S slices were excluded from the present study. The other three sections align well to specific stereotyped whisking patterns. R-C and C-C slices track circuit connections presumably active during exploratory whisking, with protraction aligning with the dorsal direction. By this alignment, M-S slices track circuit connections implicated in foveal whisking, with protraction toward the caudal direction (Knutsen et al., 2005; Petersen et al., 2020). Table 2 lists the relationships between barrels in each type of slice and the corresponding stereotyped whisking patterns and phases.

**Table 2.** Summary relating the grouping of directions

Barrel direction	Slice(s)	Possible whisking behavior relevance
Dorsal	Coronal (R-C, C-C)	Exploratory protraction
Ventral	Coronal (R-C, C-C)	Exploratory retraction
Caudal	Sagittal (M-S)	Foveal protraction
Rostral	Sagittal (M-S)	Foveal retraction

The inter-barrel relationship (first column) and slice group (second column) are linked to stereotyped animal behavior patterns (third column). Inter-barrel communication in one direction corresponds to subsequent whisker contacts or sweeping occurring in the reverse direction. These relationships and offsets are visualized in Figures 1 and 9*E*.

Basic barrel dimensions further distinguish the R-C, C-C, and M-S slices. Septal width, barrel width, and barrel height varied between these slice categories (Extended Data Fig. 1-1), providing further correspondence with inter-barrel relations. The clear boundaries highlighted by Scnn1a labeling enabled us to estimate the basic barrel dimensions. The septal width, barrel width, and barrel height, annotated on bright-field (left) and fluorescence (right) images in Extended Data Figure 1-1*A*, were estimated from the cytoarchitectural boundaries. We distinguished L2/3 from L4 based on a steep fluorescence gradient and the loss of distinction between septa and barrel hollows at the boundary. Extended Data Figure 1-1*B* compares septal widths between the R-C, C-C, and M-S slices. The mean septal width in the rostral row of R-C slices, 30.3 ± 1.5 µm (*n* = 18 septa, 15 slices), was significantly smaller than in C-C slices (51.5 ± 3.2 µm, *n* = 10 septa, 10 slices; Welch's *t* test: *t* = 5.99, *p* = 5 × 10^−5^) or in M-S slices (50.4 ± 2.8 µm, *n* = 18 septa, 13 slices, *t* = 6.38, *p* = 10^−6^). R-C and M-S septal widths did not differ significantly (*t* = 0.24, *p* = 0.8). These variations were exploited to track the transition from R-C to C-C in the sequence in which coronal slices were cut. Barrel width and height did not correlate significantly (linear regression, *R* = 0.19, *p* = 0.09), which is expected given the heterogeneity of barrel dimensions and the fact that the thickness of L4 limits barrel height to 208 ± 5 µm or less (DeFelipe et al., 2002). However, barrel and septum width showed a significant positive correlation (Extended Data Fig. 1-1*C*; *R* = 0.38, *p* = 0.01). Additionally, sagittal slices were cut at a smaller angle (less than a right angle) to the cortex layers, particularly toward the medial aspect but also in the lateral aspect. As a result, L4 is thicker in some sagittal slices and barrel height is significantly greater in M-S slices (Extended Data Fig. 1-1*D*; 159 ± 4 µm, *n* = 31 barrels) than that in R-C slices (141 ± 4 µm, *n* = 18, *t* = 3.1, *p* = 0.003) or in C-C (137 ± 4 µm, *n* = 36, *t* = 3.9, *p* = 0.0003). Barrel widths were significantly smaller in R-C slices (Extended Data Fig. 1-1*E*; 124 ± 5 µm, *n* = 18) than those in M-S slices (150 ± 6 µm, *n* = 29, *t* = 3.33, *p* = 0.002), but not significantly different from C-C slices (138 ± 6 µm, *n* = 35, *t* = 1.55, *p* = 0.13). These differences in barrel width match the trend in barrel size seen in the tangential view of BC, in which barrels become smaller in the rostro-medial aspect and have smaller cross sections in the coronal plane (Fig. 1*B*, bottom right; Petersen et al., 2020).

### Barrel responses to stimulation of different layers

**Figure 2.**
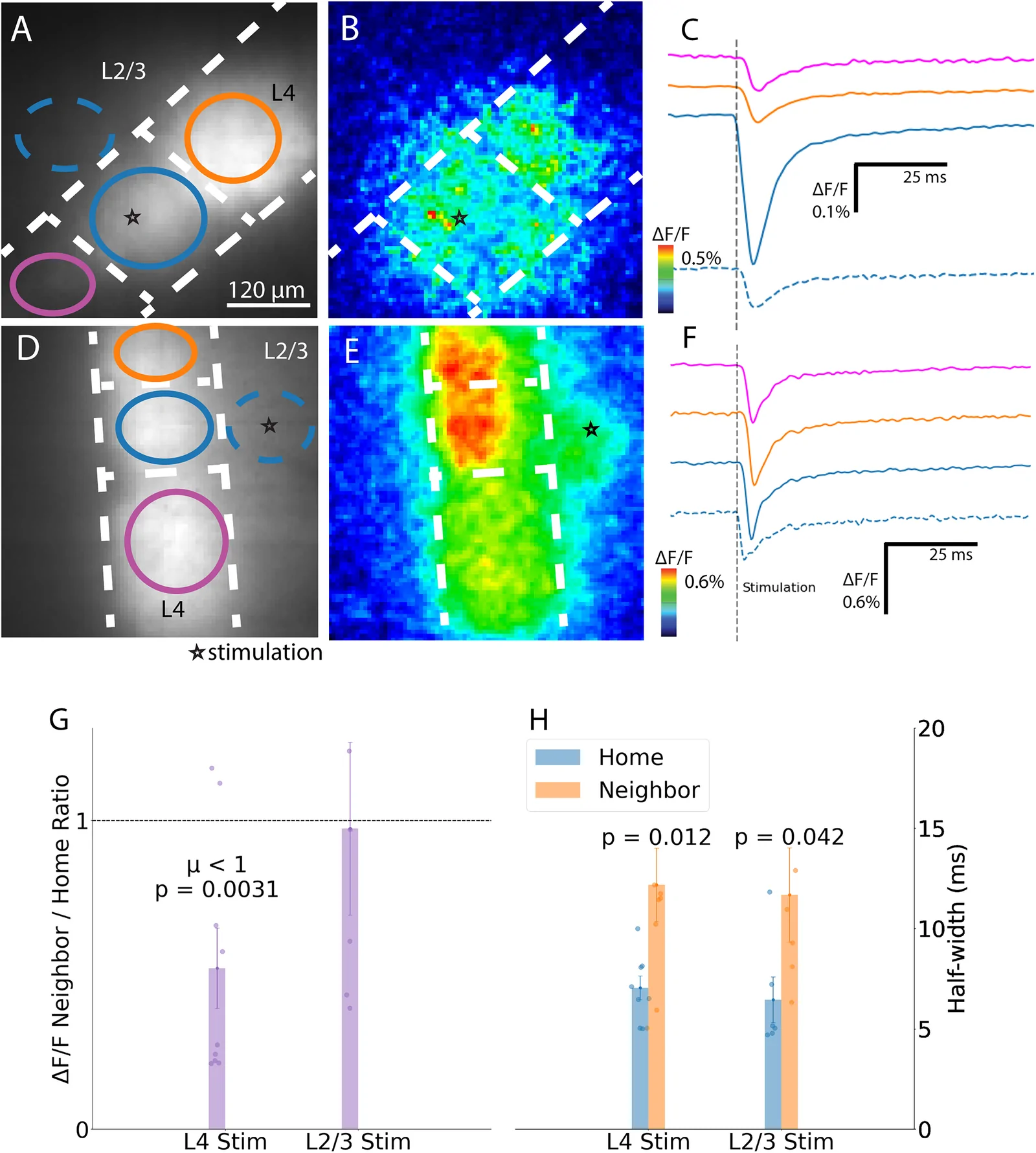
Response patterns for stimulation in L4 and L2/3. ***A–C***, L4 stimulation (current 150 µA) evokes stronger responses in home barrels than in neighbor barrels. ***A***, Image of coronal slice with star marking the stimulation site. ROIs drawn within home and neighbor barrels. ***B***, Map of peak Δ*F*/*F* (color scale denotes response amplitude; each map is scaled to its maximum). ***C***, Traces of ROI-averaged fluorescence intensity with corresponding color and line style as in ***A***. ***D–F***, L2/3 stimulation (current 200 µA) elicits responses with nearly equal amplitudes across barrels. ***D–F*** parallel ***A–C***. ***G***, Neighbor/home ratios of peak Δ*F*/*F* are significantly lower for L4 than L2/3 stimulation (*n* = 9 and 6 barrels). ***H***, Half-widths were significantly greater for neighbor barrels than home barrels for both L4 and L2/3 stimulation. Error bars show mean ± SEM with points for individual slices.

**Figure 3.**
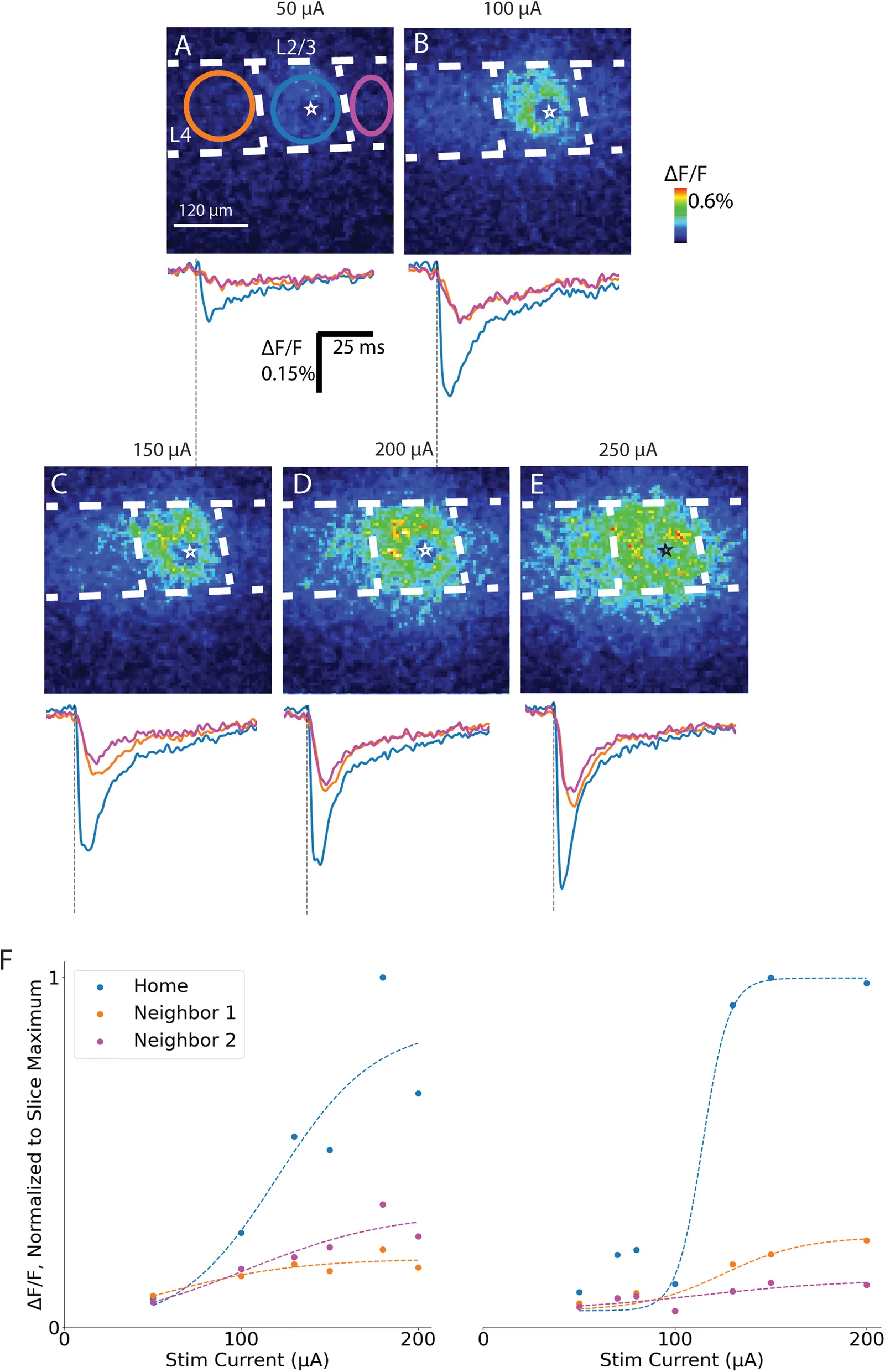
Stimulation-induced voltage changes in barrel cortex slices. ***A***, Map of peak Δ*F*/*F* in response to 50 μA stimulation in a coronal slice with stimulation site in L4 marked with a star. ROIs within home and neighbor barrels are delineated with colored boundaries. ***B–E***, Maps of peak Δ*F*/*F* in response to increasing stimulation currents (color scale denotes response amplitude normalized to the maximum in ***E***). Traces below each panel show fluorescence versus time for each ROI (colors of traces correspond with ROI outlines). Spatial scale bar in ***A*** applies to all Δ*F*/*F* maps. ***F***, Plots of peak Δ*F*/*F* versus stimulation current from two different experiments for home and neighbor barrels. Plots were fitted with sigmoid curves (see text, dotted lines) for the home and neighbor barrel plots.

Imaging hVOS signals revealed responses to stimulation in L4 (Fig. 2*A*) and L2/3 (Fig. 2*D*) in the stimulated (home) barrel as well as the immediately adjacent (neighboring) barrels. Spatial maps of color-encoded response amplitude (peak Δ*F*/*F*) illustrated that L4 stimulation elicited activity over a smaller area (Fig. 2*B*) compared with L2/3 stimulation (Fig. 2*E*). To track the evolution of responses in different regions, we selected ROIs (Fig. 2*A*,*D*) and plotted mean fluorescence within those ROIs versus time (Fig. 2*C*,*F*). Since hVOS is a negative-going voltage indicator (Chanda et al., 2005), these downward-going signals report depolarization of probe-expressing neurons. Responses near the stimulating electrode include direct, antidromic, and synaptic depolarization of Scnn1A neurons. Initial responses then spread within and beyond the stimulated barrel/layer. Stimulation of L4 with currents that produced 70%-maximal responses in L4 of the home barrel (Fig. 3*F*) produced responses with significantly smaller amplitudes in the neighbor barrels, with a Δ*F*/*F* ratio of 0.52 ± 0.13 (Fig. 2*G*; 1-sample *t* test on neighbor/home Δ*F*/*F* ratio: *t* = 3.7, *p* = 0.003, *n* = 7 animals, 9 slices). In contrast, peak Δ*F*/*F* values were indistinguishable between home and neighbor barrels when L2/3 was stimulated with similar currents; response ratio = 0.97 ± 0.28 (*t* = 0.09, *p* = 0.5, *n* = 4 animals, 6 slices). Together, these data suggest that L4 responded more strongly to L2/3 stimulation during inter-barrel communication.

Stimulating either L4 or L2/3 evoked neighbor barrel responses with significantly greater half-widths (almost twofold) than home barrel responses (Fig. 2*H*; Welch's *t* test, L4 stimulation, home: 8.8 ± 0.7, neighbor: 15.2 ± 2.3 ms, *t* = 2.7, *p* = 0.01; L2/3 stimulation, home: 8.1 ± 1.4, neighbor: 14.6 ± 2.9 ms, *t* = 2.0, *p* = 0.04). Home barrel responses tended to have a briefer half-width than neighboring barrel responses at least in part because they were closer to the site of stimulation and therefore had less dispersion of the transmitted population-level signal. The longer half-width could also reflect additional synaptic recruitment or less synaptic inhibition in inter-barrel circuitry compared with intra-barrel circuitry. We also saw small fluorescence changes in ROIs from L2/3 of the home barrel (Fig. 2*A*,*D*, dashed blue outlines; Fig. 2*C*,*F*, dashed blue traces). Because the probe is expressed in neurons with somata located in L4 (Madisen et al., 2009), we interpreted these responses as depolarization of axons and dendrites of our targeted Scnn1a neuronal subpopulation extending into L2/3.

### Spread of depolarization to multiple barrels

We characterized responses for a range of stimulation currents. In both coronal and sagittal slices (*n* = 3 animals, 8 slices), electrical stimulation in L4 elicited fluorescence changes in the home barrel and one or both neighboring barrels (Fig. 3). A coronal slice was stimulated in L4 with currents ranging from 50 to 250 μA, and maps with color-coded response amplitude (peak Δ*F*/*F*) illustrate the increase in amplitude and spatial spread of depolarization with stimulation current (Fig. 3*A–E*). Traces of fluorescence versus time from ROIs show that responses in neighboring barrels also have smaller amplitudes and slower time courses compared with the home barrel. The response amplitudes in the home barrel (blue traces) and neighbor barrels (orange and pink traces) increased with stimulation current and approached a plateau with strong stimulation.

We plotted peak Δ*F*/*F* versus current and fitted the curves to a sigmoidal function of the form $\frac{\Delta F}{F}=\;a\;(1+e^{-b(I-c)}{)}^{-1}$, with stimulation current denoted by I, and *a*, *b*, and *c* as free parameters (Fig. 3*F*, two examples). This fit converged for 7 of 8 home barrels (mean *R*^2^ = 0.93 ± 0.03) and 12 of 14 neighbor barrels (mean *R*^2^ = 0.88 ± 0.03) from 8 brain slices cut in both coronal and sagittal planes. From these fits we calculated stimulation currents for 70%-saturation of home (mean 135 ± 18, range 74–177 μA) and neighbor (mean 178 ± 33, range 81–278 μA) responses separately for each slice and used this 70% home barrel saturation current for further experiments on that slice.

### AMPA receptor blockade identifies postsynaptic responses

**Figure 4.**
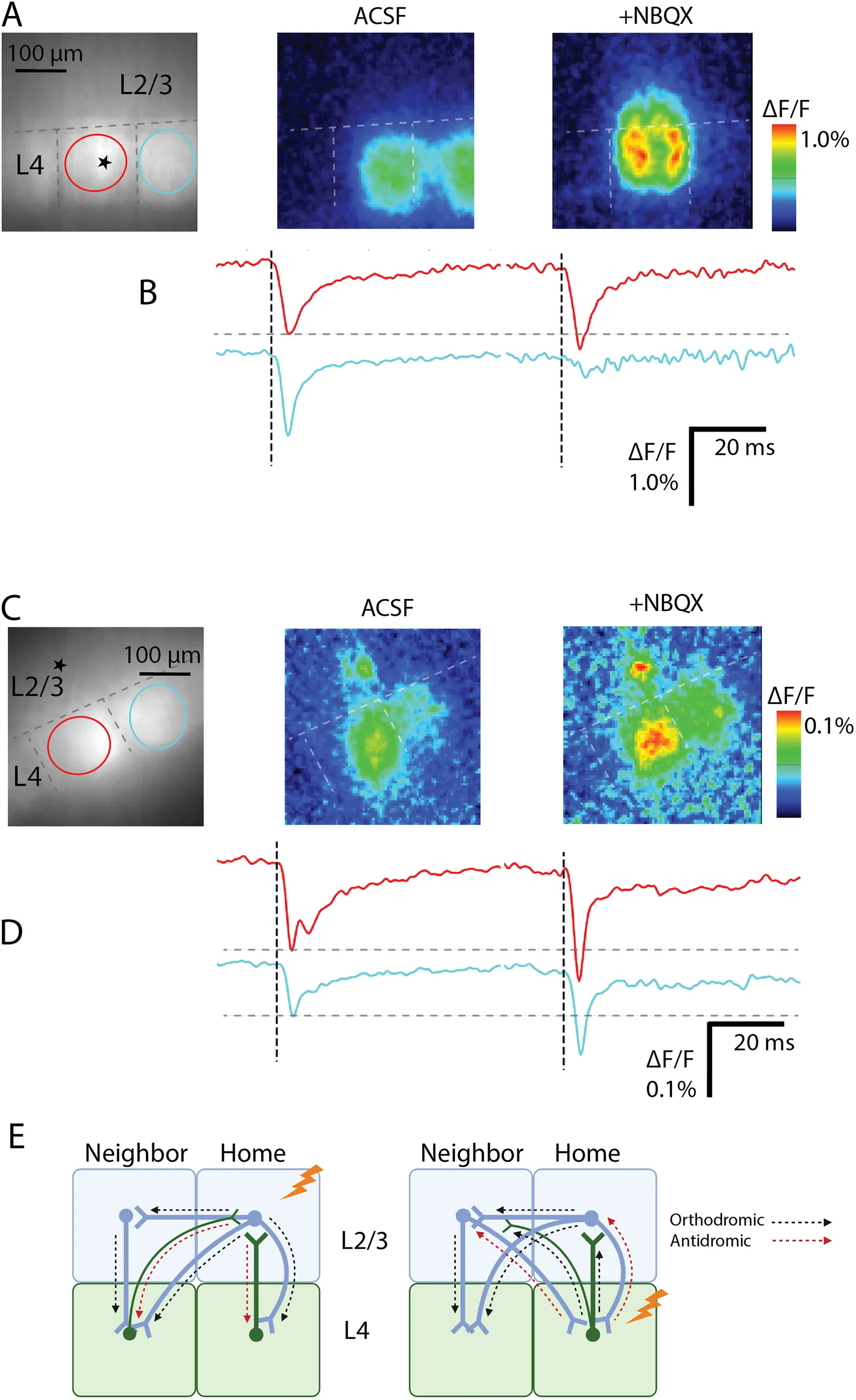
AMPA receptor blockade removes response components that depend on excitatory synaptic transmission. ***A***, L4 stimulation elicits responses in home and neighbor barrels. Left, Resting fluorescence image of a coronal slice with L4 stimulation site marked with a star and ROIs outlined. Center, Map of peak Δ*F*/*F* in control ACSF. Right, Map of peak Δ*F*/*F* after addition of 10 µM NBQX. Color scale denotes response amplitude. ***B***, Traces of fluorescence versus time for each ROI of the corresponding color in the left panel of ***A***, for control ACSF (left) and NBQX (right). Vertical black dashed line, stimulation time. Horizontal gray dashed lines aid in comparing amplitudes before and after NBQX. NBQX strongly inhibited neighbor barrel responses and slightly increased home barrel responses. ***C***, ***D***, With L2/3 stimulation, NBQX had little effect on amplitude but reduced half-width. ***C***, ***D*** parallel ***A***, ***B***. ***E***, Circuit diagram: an L4→L2/3→L4 delay mediates inter-barrel spread from L4 stimulation via monosynaptic or disynaptic pathways or both. Processes of Scnn1a neurons in L2/3 are activated antidromically by L2/3 stimulation. Thickness of axons in the diagram reflects the density of these connections in reconstruction studies (Staiger and Petersen, 2021). Orange “sparks” indicate the site of stimulation. We have included only the minimum number of relevant projections in each schematic. For example, the right diagram does not include excitatory neurons with cell bodies in the neighbor barrel because their axons are not needed to explain their inter-barrel activation by L4 stimulation (created with BioRender).

The excitation illustrated in Figures 2 and 3 reflects a combination of direct depolarization, antidromic action potentials, and orthodromic responses. Because orthodromic responses depend on excitatory synapses, we assessed their contribution by blocking AMPA receptors with the antagonist NBQX (10 μM). Figure 4*A* illustrates home and neighboring barrel responses to L4 stimulation. NBQX nearly eliminated the depolarization of the neighbor barrel (Fig. 4*A*, center and right), reducing the peak amplitude by more than fivefold in the example displayed (Fig. 4*B*, cyan trace). This strong blockade demonstrated that neighbor barrel responses depend on AMPA receptor-mediated excitatory synapses. In contrast, NBQX slightly increased average response amplitudes within the home barrel (Fig. 4*B*, red traces; amplitude ratio 1.13), suggesting these responses were largely direct and antidromic. Increases in response amplitude induced by NBQX indicated that local inhibitory interneurons, activated by excitatory synapses, limit the responses seen in ACSF alone. Reduced shunting of action potentials by AMPA receptors could also contribute to the response increase. When L2/3 was stimulated, NBQX had no inhibitory action on either home or neighbor barrel response amplitudes, indicating that antidromic action potentials dominate these responses (Fig. 4*C*,*D*).

We interpreted these results with the aid of a minimal circuit (Fig. 4*E*) in which L4 stimulation first activates excitatory neurons in L4. These neurons then excite non-Scnn1a excitatory neurons with cell bodies in L2/3, which in turn excite Scnn1a L4 excitatory neurons in the neighboring barrel. This circuit illustrates our hypothesis that intracortical multi-barrel activation uses L2/3 as a relay. Stimulation of L4 can activate neurons residing in L2/3 by excitatory transmission at L4→L2/3 synapses and/or antidromic activation of the axons of L2/3 neurons extending into L4. L2/3 neurons thus activated then excite L2/3 and L4 neurons in the neighboring barrels through the noncanonical feedback projections from lower L2/3 pyramidal neurons to L4 (Staiger and Petersen, 2021). However, we note that morphological studies have shown that L2/3→L4 projections are sparser than L6 corticothalamic projections to L4 (Staiger and Petersen, 2021), which could serve an equivalent inter-barrel communication role but could not be studied here. An additional caveat is that a few axons from L4 neurons have been reported to pass through L2/3, cross into the adjacent barrel, and then curve back down to L4. However, inter-barrel axons originating in L2/3 greatly outnumber the rare direct L4→L4 inter-barrel axons (Staiger and Petersen, 2021), consistent with the strong L4 inter-barrel signaling blockade by NBQX observed here. In contrast, L2/3 stimulation directly activates the extensive L2/3 inter-barrel arborization. These axons descend into L4 to form excitatory synapses. Finally, axons originating from L4 neurons are activated in L2/3 and conduct action potentials antidromically into L4 neurons of multiple barrels.

**Figure 5.**
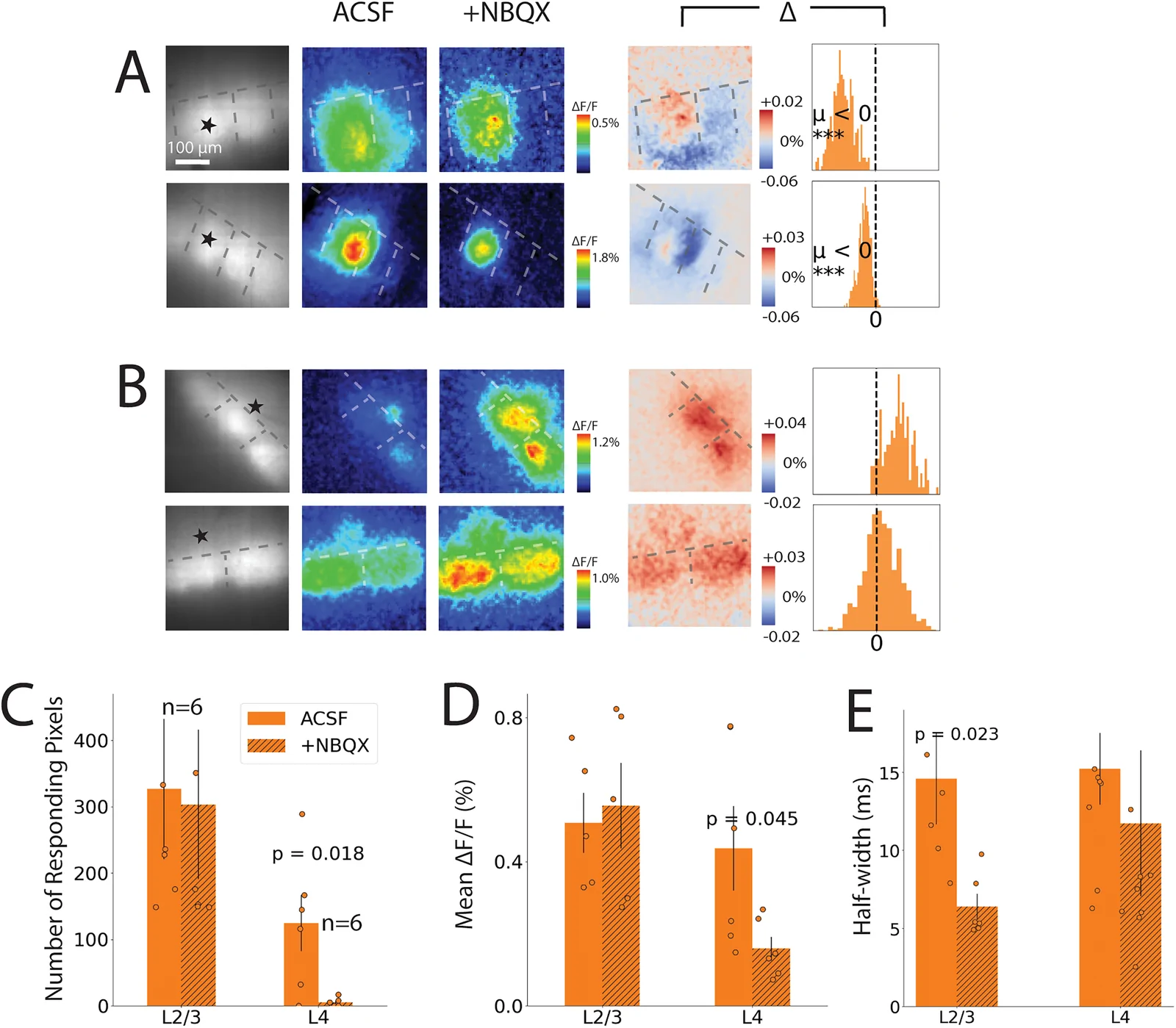
Spatial patterns of NBQX action. ***A***, ***B***, Each row corresponds to one slice before and after adding 10 µM NBQX, with L4 stimulation (***A***) or L2/3 stimulation (***B***). Left-most column, Resting fluorescence images with stimulation site (star) and layer/barrel boundaries (dashed black lines in resting fluorescent maps and dashed white lines in heatmaps). ACSF column, Maps of peak Δ*F*/*F* in control ACSF. +NBQX column, Maps of peak Δ*F*/*F* after NBQX addition. Color scale reflects the largest response in each row (union of ACSF and NBQX maps). Δ column, left, Difference maps (Δ = NBQX − ACSF) prepared by normalizing the ACSF and NBQX maps to the maximum of the union of NBQX and ACSF maps, then mapping the difference (white pixels represent zero change; red pixels represent responses increased by NBQX; blue pixels represent responses reduced by NBQX). The Δ map for L4 stimulation shows NBQX suppressed responses in the neighbor barrel L4 and enhanced responses in much of the home barrel L4. For L2/3 stimulation NBQX produced widespread enhancement. Δ column, right, histograms of pixelwise differences for neighboring barrel only. Means were significantly less than zero in both examples for L4 stimulation (*** indicates *p* < 0.001; one-sample *t* test; ***A***, top: −0.20 ± 0.03; ***A***, bottom: −0.73 ± 0.02), but greater than zero for L2/3 stimulation (***B***, top: 0.12 ± 0.06; ***B***, bottom: 0.03 ± 0.01). ***C***, Responding pixels were defined as pixels in the neighboring barrel with peak Δ*F*/*F* above a slice-specific threshold. Response area (based on number of pixels) was decreased by NBQX for L4 but not for L2/3 stimulation. ***D***. Mean peak Δ*F*/*F* was reduced by NBQX in neighbor barrels for L4 but not for L2/3 stimulation. ***E***, Half-width before and after NBQX for neighbor barrels. Error bars show mean ± SEM, with points for individual slices.

To examine spatial variations in the action of NBQX at a finer scale, we constructed difference maps comparing responses in each pixel before and after adding NBQX. We present two examples each for stimulation in L4 (Fig. 5*A*) and L2/3 (Fig. 5*B*). Difference maps (denoted with “Δ”) display the change in peak Δ*F*/*F* resulting from NBQX addition (white, no change; red, increase; blue, decrease). These difference maps show that NBQX reduced responses to L4 stimulation outside the home barrel (Fig. 5*A*, darkest blue under “Δ”), while home barrel responses were unchanged or increased. For example, in the second row/example of Figure 5*A*, the largest response in the NBQX heatmap is nearly overlapping with the stimulation location in the center of the barrel, while the largest response in the ACSF heatmap is to the right of the stimulating electrode close to the barrel edge. The responsive area decreased when NBQX was added, while the response amplitude near the stimulating electrode increased. Difference maps showed that for L2/3 stimulation NBQX broadly increased responses in L4 of both barrels (Fig. 5*B*). We also plotted differences for pixels in the neighboring barrel as histograms to the right of difference maps. Neighbor barrel distributions had means significantly less than zero for L4 stimulation (one-tailed one-sample *t* test, L4, top: *t* = −8.7, *p* < 0.001, middle: *t* = −38, *p* < 0.001, bottom: *t* = −33, *p* < 0.001) but not for L2/3 stimulation (top: *t* = 15, *p* = 1.0, middle: *t* = 9.4, *p* = 1.0, bottom: *t* = 6.1, *p* = 1.0). Nonuniform NBQX action within barrels may indicate spatial variations in local circuitry, and this could be an interesting subject for further study.

Using a slice-specific Δ*F*/*F* threshold (70th percentile of Δ*F*/*F* in ACSF), we calculated the number of responding pixels to estimate responsive area (36 μm^2^ per pixel). The area responding to L2/3 stimulation was not significantly different between NBQX and control (Welch's *t* test, *t* = 0.15, *p* = 0.88, 6 slices), while with L/4 stimulation NBQX significantly decreased response area (Fig. 5*C*; *t* = 2.8, *p* = 0.018). These plots confirm that NBQX significantly decreased peak Δ*F*/*F* of neighbor barrels for L4 stimulation (*t* = 3.1, *p* = 0.01); for L2/3 stimulation the small change was not significant (Fig. 5*D*; *t* = 0.08, *p* = 0.94).

L2/3 stimulation in ACSF elicited responses in L4 that were broad and sometimes biphasic (Fig. 4*D*), suggesting that synaptic delays of several milliseconds separated synaptic responses from antidromic responses. Since L4→L2/3 projections outnumber L2/3→L4 projections (Staiger and Petersen, 2021), we expect that the antidromic component will dominate L4 responses to L2/3 stimulation, while synaptic components, which are slower, will make responses broader. Response half-width is sensitive to these two components, and in neighboring barrels NBQX reduced half-widths of responses to L2/3 stimulation by more than twofold from 14.6 ± 2.9 to 6.4 ± 0.8 ms (Fig. 5*E*; Welch's *t* test, *t* = 2.7, *p* = 0.02). In contrast, responses to L4 stimulation do not appear biphasic in ACSF (Fig. 4*B*). NBQX produced only a small reduction in half-width of neighboring barrel responses from 15.2 ± 2.3 to 11.7 ± 4.7 ms, which was not significant (Fig. 5*E*; *t* = 0.7, *p* = 0.5). This indicates that antidromic action potentials cannot be detected and that small incompletely blocked synaptic potentials determine the half-widths of these responses.

### Propagation through layers and barrels

**Figure 6.**
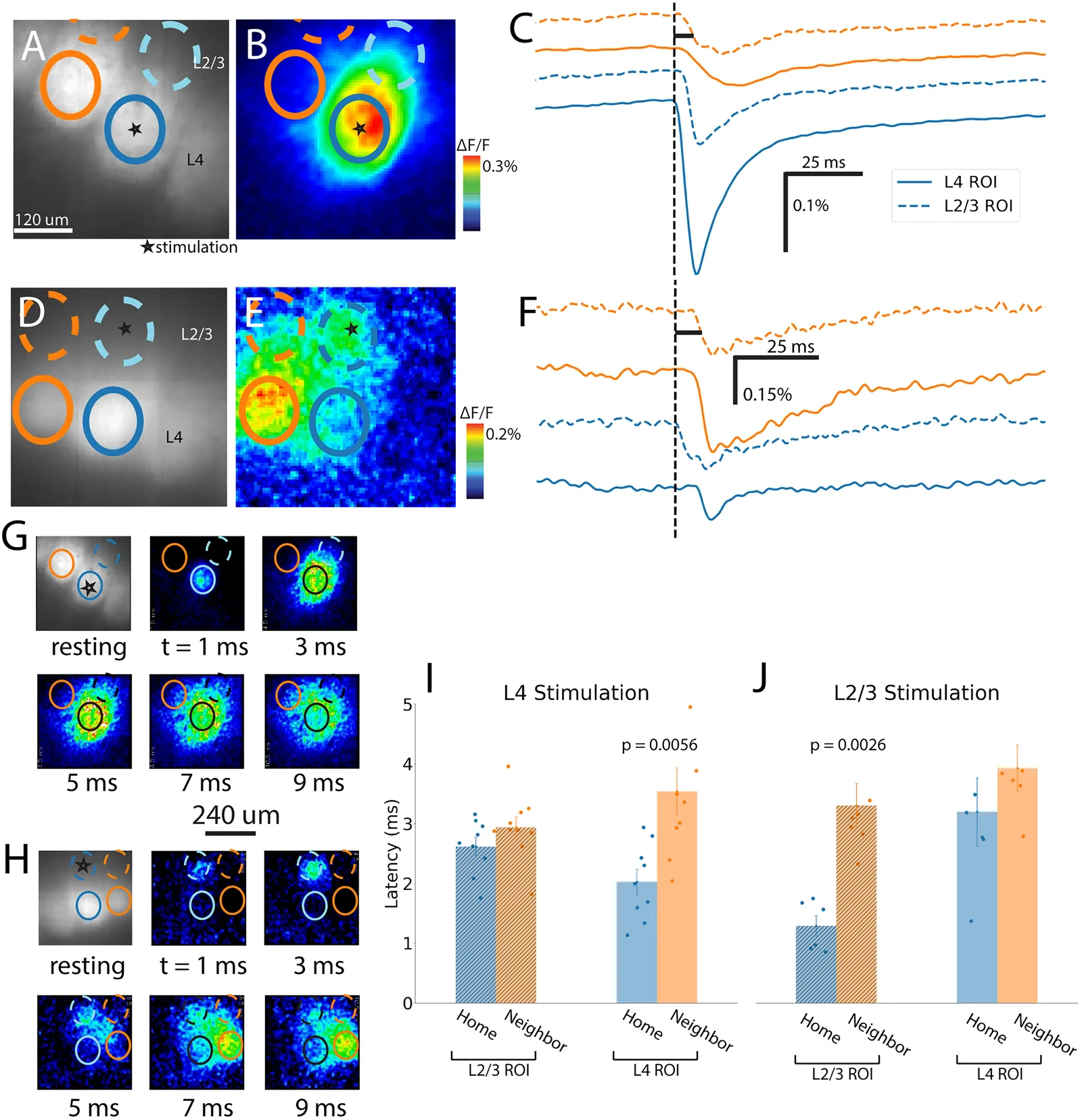
Spatiotemporal spread of excitation across barrels and layers. ***A***, ***D***, Fluorescence images showing ROIs and stimulation sites. ***B***, ***E***, Heat maps of peak responses (color scale denotes amplitude). ***C***, ***F***, Traces in the home (blue) and neighbor (orange) barrels of L4 (solid) and L2/3 (dotted). ROI outlines in maps and traces are in corresponding colors. Dashed black line marks stimulation time. With L4 stimulation, the response in the L4 home barrel precedes that in L2/3 and in neighbor barrels (Movie 1). ***D–F***, Stimulation in L2/3 elicits near-simultaneous activation in ROIs of the three nearby locations following responses in the stimulated L2/3 ROI (Movie 2). ***G***, ***H***, Top-left, Fluorescence images corresponding to ***A*** and ***D***, with successive panels providing a time-lapse sequence of Δ*F*/*F* maps at 2 ms intervals following stimulation in L4 (***G***) or L2/3 (***H***) to illustrate the spread of excitation over a 9 ms window. ***I***, ***J***, Latency to half-maximum Δ*F*/*F* for each ROI averaged over multiple slices for L4 stimulation (*n* = 10) and L2/3 stimulation (*n* = 7). Means are plotted ± SEM with points for individual slices. L4 stimulation leads to sequential activation of L4 in home followed by neighbor barrels, while L2/3 stimulation leads to more synchronous activation across regions.

We further tested the hypothesis that inter-barrel L4→L4 communication depends on conduction through L2/3 by following the propagation of responses along this route over time. This is made possible by the signals in Scnn1a-expressing neuronal processes extending into supragranular layers (Fig. 2). For the slice with resting fluorescence shown in Figure 6*A*, and peak response map shown in Figure 6*B*, the traces in Figure 6*C* illustrate this sequence. Responses in L4 of the home barrel are followed first by responses in L2/3 of the home barrel and then by responses in L4 of the neighboring barrel. Stimulating L2/3 (Fig. 6*D*) produced the response heatmap in Figure 6*E*, and the traces in Figure 6*F* indicate nearly simultaneous activation of L4 in the home barrel (solid blue trace) along with L4 and L2/3 in the neighboring barrel (solid and dotted orange traces).

Amplitude (Δ*F*/*F*) maps at regular time intervals chart the spread of excitation evoked by stimulation of L4 (Fig. 6*G*) and L2/3 (Fig. 6*H*). Note that these maps are a sequence of snapshots in time, as opposed to peak amplitude maps displayed in the preceding figures. The time from stimulation to half-maximum on the rising phase, or latency, was 1.9 ms for L4 home barrel responses to L4 stimulation, followed by the L2/3 home barrel at 3.0 ms and the L2/3 neighbor barrel at 3.9 ms (Fig. 6*G*). L4 of the neighbor barrel responded with the longest latency (6.2 ms), reflecting synaptic activation of L2/3 excitatory neurons, conduction through their axons, and synaptic activation in L4. We were not able to distinguish between monosynaptic and disynaptic signaling based on latency. With L2/3 stimulation, local responses in L2/3 appear first (1.5 ms), followed within 3–4 ms by essentially simultaneous activation of the other three regions (appearing as faint blue), starting in the 5 ms snapshot (Fig. 6*H*).

Movie 1 presents another experiment of spread of responses to L4 stimulation, illustrating the L4→L2/3→L4 activation sequence. Movie 2 presents a different example with L2/3 stimulation, illustrating that L2/3→L4 antidromic conduction arrives in both barrels nearly simultaneously, with a second synaptic wave creating a biphasic response (Fig. 4*D*, traces). Mean latencies also support these sequences for L4 (Fig. 6*I*, *n* = 10) and L2/3 stimulation (Fig. 6*J*, *n* = 7). Latencies differed significantly among responses at the four locations (ANOVA; Fig. 6*I*, *F* = 6.3, *p* = 0.0017; Fig. 6*J*, *F* = 7.9, *p* = 0.0010). Due to propagation through L2/3, the latency in L4 differed significantly between home and neighbor barrels with L4 stimulation (*t* = 3.4, *p* = 0.006), but not with L2/3 stimulation (*t* = 1.1, *p* = 0.32). L2/3 response latencies were not significantly different between home and neighbor barrels (L2/3 stimulation: *t* = 4.6, *p* = 0.0026; L4 stimulation: *t* = 1.5, *p* = 0.17) as these locations are closely spaced along the hypothesized L4→L2/3→L4 relay. The general trend of increasing latencies for responses in L4 of the home barrel, to L2/3 of the home barrel, to L2/3 of the neighbor barrel, to L4 of the neighbor barrel is corroborated in Movie 1 and is consistent with the hypothesis that electrical activity takes this route during inter-barrel communication.

**Movie 1.** Spatiotemporal spread of excitation across barrels and layers for L4 stimulation. Left, Animated sequence of heat maps of peak responses from just before stimulation to 10 ms poststimulation, paralleling the heatmap time series of Figure 6*G* for a separate example. The resting fluorescence image is shown at the animation end. Stimulation site is marked with a star. Right, Traces in the home (L4: blue, L2/3 green) and neighbor (L4: orange, L2/3: red) barrels. ROI outlines of the same color in maps correspond to traces. Dotted blue line marks stimulation time. With L4 stimulation, the response in L4 of the home barrel precedes that in L2/3 and in neighbor barrels. [View online]

**Movie 2.** Stimulation in L2/3 shows near-simultaneous activation of three unstimulated ROIs. Left, Heat maps of peak responses animated from just before stimulation to 10 ms poststimulation, paralleling the heatmap time series of Figure 6*H* for a different slice. The resting fluorescence image is shown at the animation end. Stimulation site is marked with a star. Right, Traces in the home (L4, blue; L2/3, green) and neighbor (L4, orange; L2/3, red) barrels. ROI outlines of the same color in maps corresponding to traces. Dotted blue line marks stimulation time. Biphasic responses reflect multiple L2/3-L4 pathways mediating inter-barrel communication. [View online]

### Intra- and inter-barrel velocity

**Figure 7.**
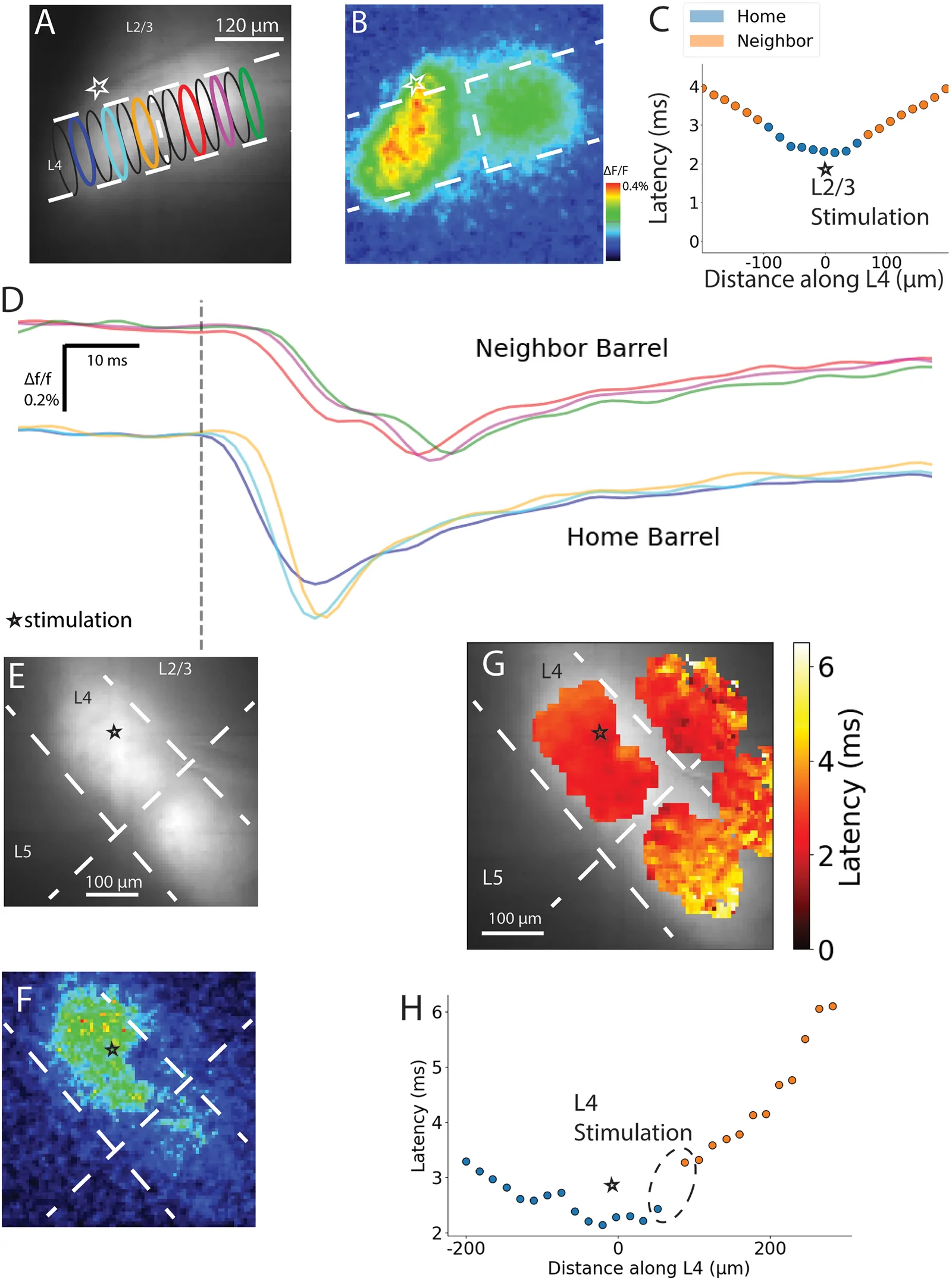
Velocity of propagation into neighbor barrels. ***A***, Fluorescence image of a coronal slice showing ROIs used to compute velocity in home and neighbor barrels. ***B***, Peak Δ*F*/*F* response map of the slice shown in ***A*** with stimulation site and layer/barrel boundaries annotated. ***C***, Latency versus distance along L4 for responses to L2/3 stimulation, from a different slice from that in ***A***. Blue points are for the home barrel, while orange points are for neighbor barrels on either side. Segments of constant slope were used to determine velocity (see Materials and Methods). ***D***, Δ*F*/*F* traces from home and neighbor barrel ROIs of the corresponding colors in ***A***, from sites arranged along the direction of propagation. ***E***, Fluorescence image of a sagittal slice overlaid with the latency map, with the L4 stimulation site marked with a star. ***F***, Peak Δ*F*/*F* response map with stimulation site and layer/barrel boundaries annotated for the slice shown in ***E***. ***G***, Heatmap of latency for the slice in ***E***, laid over the resting fluorescence image. Latencies are shown only within the barrel ROIs, where fluorescence emanating from the hVOS probe and thus responses are concentrated. ***H***, ROIs from L4 in the example in ***E–G*** were used to plot latency versus distance along L4. Latency jumps abruptly across the home-neighbor inter-barrel boundary at the septum (black dashed ellipse). See Extended Data Figure 7-1 for comparison of velocity across stimulation sites and home versus neighbor barrels.

Figure 6, *G* and *H*, illustrates the increase in response latency with distance along the pathways traced by axons. We used these sequences to estimate conduction velocity, constructing ROIs 18–30 μm wide within L4 to track intralaminar conduction within and between barrels (Fig. 7*A*, 30-µm-wide ROIs). The heatmap in Figure 7*B* shows responses to L2/3 stimulation that spread to L4 of the neighboring barrel. Plotting latency versus distance for the ROIs in L4, we find smooth propagation of responses evoked by L2/3 stimulation (Fig. 7*C*). Figure 7*D* displays traces from the home and neighbor barrels in Figure 7*A*. With L4 stimulation (sagittal; Fig. 7*E*, resting fluorescence; Fig. 7*F*, Δ*F*/*F* heatmap), we mapped latency color-coded as red-to-yellow overlaid on the fluorescence image (Fig. 7*G*) and observed a gradient, particularly across the L4 neighbor barrel.

10.1523/ENEURO.0109-26.2026.f7-1 figure 7-1 **Velocity and half-width persist across manipulations. A**. Boxplot and scatter of velocity before and after NBQX. NBQX had no significant effect. **B.** Velocity was greater in the home barrel but not significantly different from neighbor barrel velocities. **C.** No significant change in velocity between the first and second responses. **D.** Response half-width was also unchanged. Note that the difference between neighbor and home half-widths was discussed in regard to Fig. 3H, so we have left it unmarked here to avoid repetition of results and to focus on the present comparison of first and second responses. Boxplots show minimum, first quartile, median, third quartile, and maximum with scatter; means ± SEM reported in Results. Statistics used barrel means as independent samples, although individual ROI measurements are displayed. The mean velocity within L4 neighbor barrels was similar regardless of stimulation site (open circle markers; L2/3: 129 ± 12 µm/ms, L4: 122 ± 5 µm/ms, T = 0.98, p = 0.36). L4 neighbor velocities were computed for the minority of cases where NBQX did not completely abolish responses (ACSF: n = 31 responding L4 neighbor barrels, NBQX: n = 6). NBQX had no significant effect on velocity (Fig. S3A, bordered markers; ACSF and NBQX, L2/3: T = 1.16, p = 0.27, L4: T = 1.0, p = 0.34). Depression in amplitude was not accompanied by a change in half-width (Fig. S2C, home: T = 0.14, p = 0.89; neighbor: T = 0.66, p = 0.51). Additionally, there was no change in conduction velocity from the first to second pulse (Fig. S2D, 99 ± 9 μm/ms to 98 ± 8 μm/ms, T = 0.11, p = 0.91), suggesting a constant speed for processing information between adjacent barrels. Download figure 7-1, TIF file.

In Figure 7*H*, a plot of latencies along L4 (orange) shows a jump of 0.60 ms over an 18 μm distance at the barrel boundary (black dotted ellipse), compared with the mean increment of 0.21 ± 0.04 ms per 18 μm in the rest of this plot. Such jumps were not seen for responses to L2/3 stimulation (Fig. 7*C*). However, other slices showed accelerations rather than delays in their L4 trajectories or delays occurring within the neighboring barrel. These qualitative boundary changes in velocity mark discrete transitions from activation of the home barrel through direct depolarization, action potentials, and synaptic potentials, to activation of the neighboring barrel through parallel paths of inter-barrel communication that depend on synaptic signaling through L2/3 (Fig. 7*C*). Extended Data Figure 7-1 compares velocity across stimulation sites and home versus neighbor barrels.

### Inter-barrel communication biases

**Figure 8.**
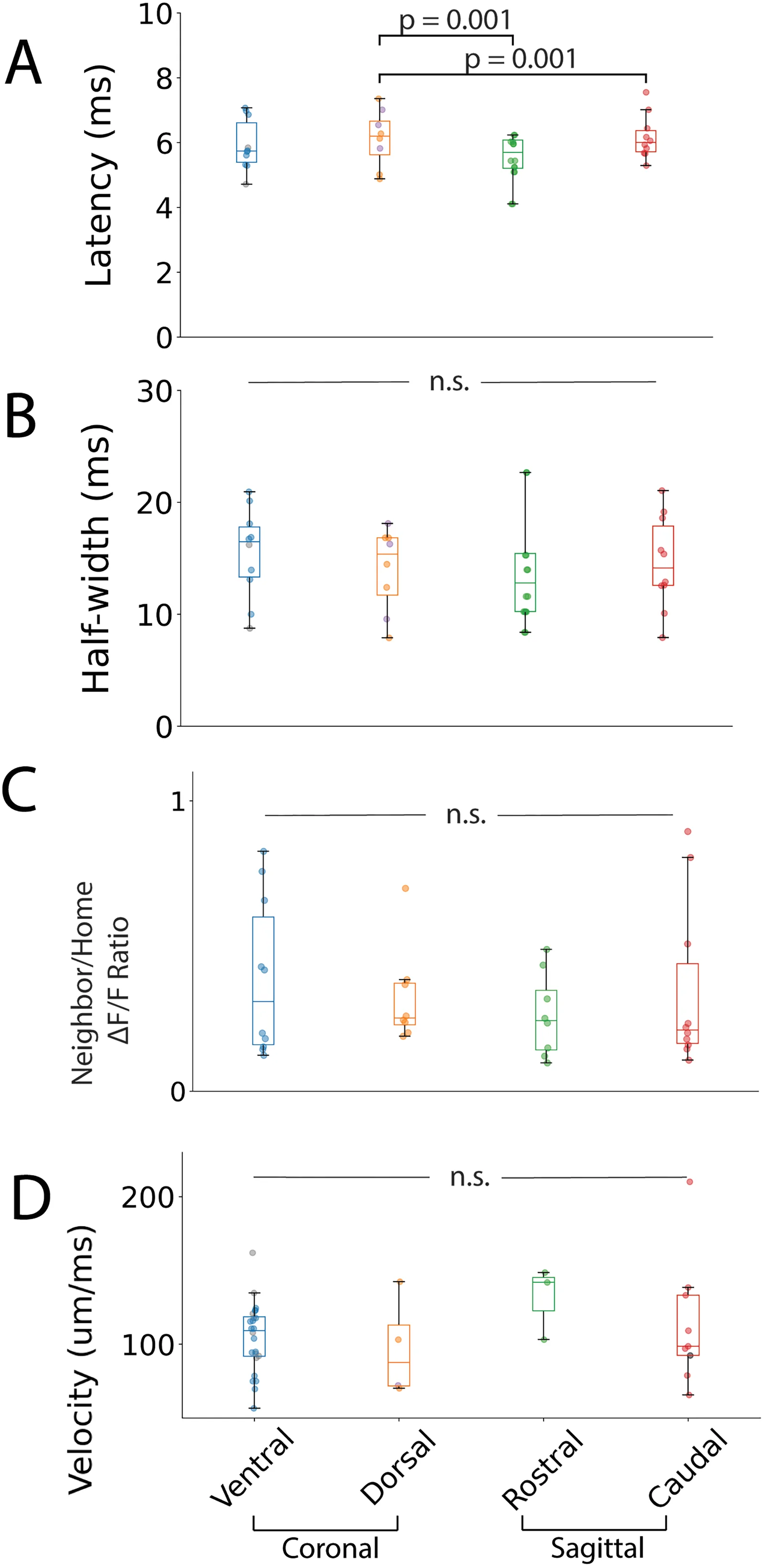
Comparison of responses between propagation directions. Latency (***A***), half-width (***B***), neighbor/home Δ*F*/*F* ratio (***C***), and velocity (***D***) of neighbor barrel responses grouped by propagation direction: rostral, caudal, dorsal, or ventral relative to the home barrel (Table 2). Colors of points: red, caudal in M-S slices; green, rostral in M-S slices; purple, dorsal in C-C slices; gray, ventral in C-C slices; orange, dorsal in R-C slices; and blue, ventral; R-C and C-C of the same direction are grouped in boxplots and scatter plots. Boxplots show minimum, first quartile, median, third quartile, and maximum, with points for individual slices; means ± SEM reported in the Results. Statistical analysis used one-way ANOVA and Tukey's HSD as appropriate.

We compared inter-barrel communication in coronal (Fig. 1*A*,*B*) versus sagittal (Fig. 1*C*,*D*) slices in both directions with the goal of identifying directional biases in intracortical microcircuitry that could potentially contribute to the processing of sensory input (Table 2). Successive whisker contacts with a fixed object occur several milliseconds apart, opposite to the direction of whisking. In particular, in sagittal slices barrels map onto whiskers along the rostro-ventral direction toward which foveal protraction occurs, while barrels in coronal slices map onto rostrodorsal whisking, presumably relevant to exploratory protraction. We determined how inter-barrel signaling varies in circuits relevant to specific directions or phases. Figure 8 compares barrel-averaged latency, half-width, neighbor/home Δ*F*/*F* ratio, and within-barrel conduction velocity across four directions (combining data from R-C and C-C slices).

Response latencies observed here were comparable to the time between successive whisker contacts or sweeps through the same space and varied across directions (caudal: 6.2 ± 0.2 ms; rostral: 5.8 ± 0.4 ms; dorsal: 6.1 ± 0.3 ms; ventral: 5.9 ± 0.3 ms). These differences were significant (Fig. 8*A*; ANOVA, *F* = 5.9, *p* = 0.001, *n* = 6 coronal, 5 sagittal animals; *n* = 10, 8, 8, 10 slices), with caudal > dorsal (Tukey's HSD: *p* = 0.001) and dorsal > rostral (*p* = 0.001). This indicates that the speed of communication between barrels is anisotropic.

In contrast, anisotropy could not be detected in response half-width or amplitude. Mean half-widths of neighbor barrel responses (Fig. 8*B*; caudal: 14.6 ± 1.3 ms; rostral: 13.5 ± 1.6 ms; dorsal: 14.0 ± 1.3 ms; ventral: 15.5 ± 1.3 ms) were indistinguishable across directions (ANOVA, *F* = 0.5, *p* = 0.77, *n* = 10, 8, 8, 10, respectively). We normalized each neighbor barrel Δ*F*/*F* to its home barrel Δ*F*/*F* (Fig. 8*C*; caudal: 0.35 ± 0.09; rostral: 0.26 ± 0.05; dorsal: 0.32 ± 0.06; ventral: 0.39 ± 0.09) and found no anisotropy in the Δ*F*/*F* ratios (ANOVA, *F* = 0.45, *p* = 0.72).

Figure 8*D* compares propagation velocity within L4 of neighboring barrels in response to L4 stimulation. Direction did not significantly impact velocity (ANOVA, *F* = 0.69, *p* = 0.56; ventral: 104 ± 5 µm/ms, *n* = 19; caudal: 113 ± 14 µm/ms, *n* = 9, dorsal: 97 ± 17 µm/ms, *n* = 4; rostral: 131 ± 14 µm/ms, *n* = 3). While in vivo studies showed that deflection-evoked signals travel faster along rows than arcs (Petersen et al., 2003), and whisking velocity is greater along the rostral-caudal axis (Petersen et al., 2020), these velocities depend on many parts of the nervous system while our velocities in slices reflect local circuitry within the cortex. Although traversing wider barrels in the caudal aspect of BC (Fig. S1*D*,*E*) could require faster conduction, we did not see a significant difference. Conduction velocity, half-width, and Δ*F*/*F* of responses to single-pulse stimulation were the same in all directions and thus isotropic. In contrast, inter-barrel latency varied significantly between directions and was thus anisotropic. Our examination of short-term plasticity also revealed anisotropy (following section).

### Paired-pulse depression

**Figure 9.**
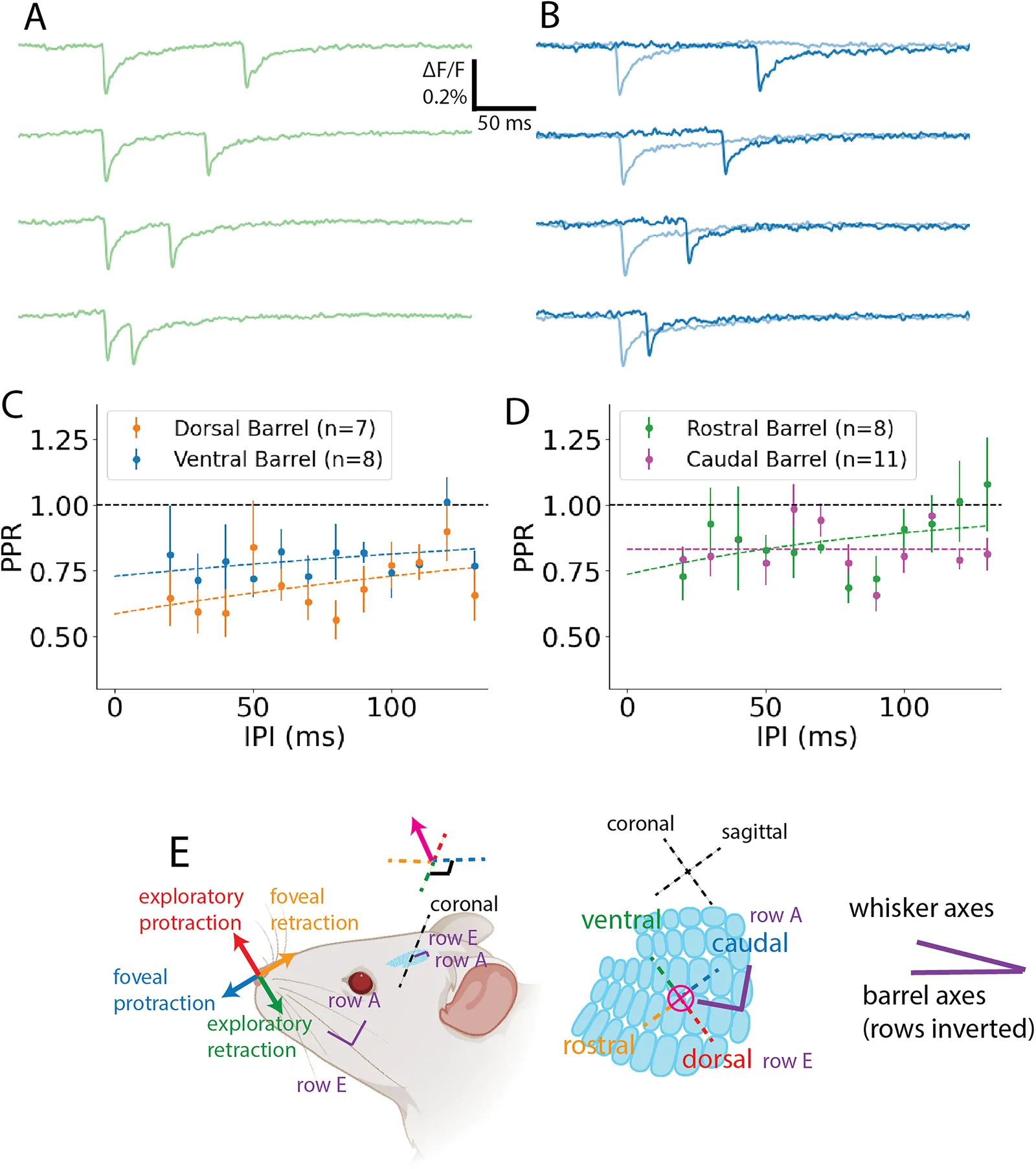
Paired-pulse depression and ratios in neighbor barrels across directions. ***A***, Fluorescence traces from barrel delimited ROIs in L4 for paired-pulse stimulation at four different interpulse intervals (IPIs). At small IPIs, the first response does not return to baseline before the second pulse, leading to temporal summation that increases the second response. ***B***, Single-pulse control responses are recorded (light blue traces) immediately before or after (determined randomly) the paired-pulse responses. Subtracted traces (dark blue), computed as paired-pulse minus single-pulse, remove temporal summation. ***C***, ***D***, Paired-pulse ratio (PPR, the amplitude of the second response divided by the first) plotted as a function of IPI for each direction, for coronal (***C***) and sagittal (***D***) slices. Solid curves represent exponential fits to recovery (see text). ***E***, Diagram relating the kinematics of whisking patterns to barrel layout, showing that the dorsal-ventral plane in coronal slices tends to align with exploratory whisking, and the rostral-caudal plane in sagittal slices aligns with foveal whisking (Knutsen et al., 2005; Petersen et al., 2020). Orientation of four directions is based on the Brain Globe 3D atlas of barrel cortex viewed in coronal and sagittal planes (Claudi et al., 2020). The schematic shows row-inversion, elevation, and azimuthal tilt to map whiskers onto their contralateral barrels. The pink arrow and pink circle with x indicate the orientation of the normal vector out of BC plane (circle with an x in it represents a vector pointing away from the viewer). Colors red, green, orange, and blue show the association between whisking pattern/phase and barrel field direction. Table 2 lists this mapping. Created with BioRender (***E***).

Short-term synaptic plasticity shapes the processing of repetitive inputs. We investigated short-term plasticity of inter-barrel communication by stimulating L4 with paired pulses and evaluating the PPR of responses in L4 of neighboring barrels. Temporal summation was evident when the interpulse interval (IPI) was <80 ms (Fig. 9*A*) so we subtracted single-pulse controls from the paired-pulse responses (Fig. 9*B*, dark blue traces). The subtracted traces revealed paired-pulse depression and a slow, incomplete recovery over IPIs from 20 to 120 ms. Following up on our examination of directional biases in inter-barrel communication, we plotted PPR versus IPI for neighboring barrel responses in the four different barrel field directions (Fig. 9*C*,*D*). PPR had a significant dependence on IPI [2-way ANOVA, PPR ∼ IPI + C(Direction) + IPI:C(Direction), *F* = 5.3, *p* = 0.021] and additionally varied with direction (*F* = 3.2, *p* = 0.012).

We fitted these PPR versus IPI plots to the function $\mathrm{PPR}=1-ae^{\frac{-\mathrm{IPI}}{\tau }}$ to determine the recovery time constant, *τ*, and intercept at IPI = 0 ms, 1−a. The qualities of the fits were acceptable for dorsal (*R*^2^ = 0.20), ventral (*R*^2^ = 0.14), and rostral (*R*^2^ = 0.28), but poor for caudal (*R*^2^ = 0.002). Table 3 summarizes the intercepts and recovery time constants, which we compared across the same four directions examined in the preceding section. All four directions show significant depression, with PPR intercepts significantly <1 (dorsal: *t* = 5.6, *p* = 0.0006, ventral: *t* = 5.4, *p* = 0.0009, caudal: *t* = 3.2, *p* = 0.005, rostral: *t* = 3.1, *p* = 0.009). The intercept varied significantly (ANOVA, *F* = 8.7, *p* = 0.0003) and was lowest in the dorsal direction (Tukey's HSD: caudal > dorsal, *p* = 0.001; rostral > dorsal, *p* = 0.032; ventral > dorsal, *p* = 0.025).

**Table 3.** The PPR intercept at 0 ms and the exponential recovery time constant *τ* for each direction of inter-barrel propagation, determined by fitting the equation in the text to the plots in Figure 9

	PPR intercept, 1-a	Recovery time constant *τ* (ms)	*n*
Dorsal	0.58 ± 0.08	235 ± 150	7
Ventral	0.73 ± 0.06	269 ± 230	8
Caudal	0.83 ± 0.06	ND	8
Rostral	0.73 ± 0.12	110 ± 85	11

The caudal direction did not show measurable recovery within the 120 ms IPI testing range, so *τ* was not determined. Values presented as mean ± SEM (*n*, number of slices).

Direction also significantly impacted recovery from depression (ANOVA, *F* = 8.2, *p* = 0.002). Although dorsal and ventral barrel *τ* values were indistinguishable (Tukey's HSD: *p* = 0.9), they were twice the rostral value (rostral vs ventral, *p* = 0.053; dorsal vs rostral, *p* = 0.15). In contrast, as suggested by the failure of the exponential fit, among the various directions caudal barrels do not show significant recovery within this range of IPIs and show the least depression (Fig. 9*C*). The illustration in Figure 9*E* relates the directions between barrels to the angular offset of the whisking field, showing the whisking phase relevant to each BC slice plane. This highlights the correspondence of caudal and dorsal directions to foveal and exploratory protraction, respectively, and of rostral and ventral directions to foveal and exploratory retraction, respectively. The distinction between foveal and exploratory is most relevant near the fully protracted whisker setpoint, whereas whisker elevation is more constant near the fully retracted setpoint (Knutsen et al., 2005).

Figure 9*E*, left and center, illustrates the rotation from the whisker to contralateral barrel field, which inverts rows (Wu et al., 2011), as well as the ∼8° downward tilt in elevation and ∼22° horizontal tilt (Sumser et al., 2017). The pink arrow in the left schematic represents the normal vector coming out of the barrel field plane; in the center schematic, the circle with an x in it signifies that this normal vector is pointing into the page. Finally, because inter-barrel communication in one direction corresponds to activity traveling to meet a subsequent contact of whisking in the opposite direction, the protraction and retraction directions (Fig. 9*E*, left, red, green, blue, and orange) are flipped from their corresponding barrel field directions (Fig. 9*E*, center, dorsal, red; ventral, green; caudal, blue; rostral, orange). These relationships are enumerated in Table 2. The coronal and sagittal sections are invariant to the 22° horizontal rotation, but the 8° elevation angle offset between barrel field and whisker field affects these sections (Fig. 9*E*, right). Foveal and exploratory whisking patterns also do not cleanly map onto row- or arc-based directions. During free whisking exploratory protraction may introduce elevation angle increases of as much as 0.3° of upward movement per degree of forward movement, for B and C rows (Knutsen et al., 2008). The maximal retraction to maximal protraction span of 105° results in an elevation change of 32° for these rows.

## Discussion

In this study we imaged evoked voltage changes specifically in Scnn1a-expressing excitatory neurons in mouse BC slices to characterize inter-barrel communication in isolation from thalamic or inter-areal inputs. The cell bodies of Scnn1a neurons reside in L4. They are the primary excitatory targets of thalamic inputs and are depolarized during whisking and object touch (Kiritani et al., 2023). Our experiments suggest that these neurons can communicate with their counterparts in adjacent barrels using an excitatory synaptic relay that passes through L2/3. Optogenetic silencing of L2/3 would provide a critical test of this hypothesis. Inter-barrel signaling had latencies comparable to the latencies of successive whisker contacts during active whisking, and the longest latency was in the caudal direction. In contrast, neighbor barrel conduction velocity, half-width, and amplitude ratio did not vary between directions and thus were isotropic. Paired-pulse depression was asymmetric, with greatest depression in the exploratory protraction direction. These findings reveal direction-, frequency-, cell type-, and layer-specific mechanisms that can shape somatotopic information flow in the BC.

### Mechanistic insights into inter-barrel signaling

L4 stimulation elicited AMPA receptor-dependent responses in L4 of neighboring barrels. The functional independence of barrels in L4 is an important concept in cortical processing (Petersen and Sakmann, 2001; Laaris and Keller, 2002; Sato et al., 2008; Hill and Greenfield, 2014), but communication between barrels, and more broadly between columns, is essential to the integration of sensory information (Petersen et al., 2003; Narayanan et al., 2015; Jancke, 2017; Roe, 2019). Petersen and Sakmann (2001) stimulated BC slices at the base of L4 (to activate thalamocortical inputs to L4) with currents in the low end of the range used here and did not observe spread to L4 in neighbor barrels. Sato et al. (2008) used stronger stimulation in the center of the barrel in L4, and in the presence of bicuculline their voltage-sensitive dye imaging revealed spread to L4 of neighboring barrels, similar to the present observations. We used a voltage sensor expressed in excitatory neurons to reveal clear activation of L4 in neighboring barrels without blocking inhibition. However, maximal response amplitudes in neighboring barrels remained below those in the home barrel. The requirement of higher stimulation currents to induce spread to L4 of neighbor barrels (Fig. 3) suggests that this inter-barrel pathway has a higher threshold than local intra-barrel activation.

AMPA receptor blockade confirmed that the neighbor barrel responses in L4 were synaptic. Multiple inter-barrel pathways between L4 can be envisioned to generate the sequence of activation we observed (Fig. 4*E*): these include $L4^{\mathrm{home}}\to L2/3^{\mathrm{home}}\to L2/3^{\mathrm{neighbor}}\to \;L4^{\mathrm{neighbor}}$; or $L4^{\mathrm{home}}\to L2/3^{\mathrm{home}}\to \;L4^{\mathrm{neighbor}}$; with lesser contributions from $L4^{\mathrm{home}}\to L2/3^{\mathrm{neighbor}}\to \;L4^{\mathrm{neighbor}}$. Stimulating in L2/3 robustly activated L4 of multiple barrels. NBQX did not block these responses, suggesting they are predominantly antidromic. This provided evidence for L4→L2/3 connections crossing between barrels within L2/3, consistent with morphological findings (Feldmeyer et al., 2002; Narayanan et al., 2015; Staiger and Petersen, 2021). The $L2/3^{\mathrm{home}}\to L2/3^{\mathrm{neighbor}}$ pathway could not be observed directly in the present study as Scnn1a does not label neurons with cell bodies in L2/3, but this pathway may contribute to the component of Scnn1a-expressing neuron synaptic responses to L2/3 stimulation that arrive at a longer latency and were abolished by NBQX (Fig. 4*D*). Infragranular layers may also contribute to inter-barrel signaling, particularly with L6 corticothalamic pyramidal neurons furnishing both inter-barrel connections and projections to L4 (Staiger and Petersen, 2021). However, in the present study we focused on L4 and L2/3.

With both L2/3 and L4 stimulation, NBQX revealed an inhibitory action: increased response amplitudes suggested that AMPA receptor-mediated activation of interneurons engages feedforward inhibition within and between barrels. The NBQX-induced response increases may also reflect another mechanism such as removal of AMPA receptor shunting of action potentials. To test the role of feedforward inhibition directly, we have attempted experiments with 5 μM GABAzine, but stimulation currents of only 50 μA elicited epileptiform-like barrages lasting 200 ms or more (data not shown). Determining the different contributions to the response increases produced by NBQX will require additional experiments with varying concentrations of NBQX and GABAzine.

### Intracortical velocity

Our velocities of 104–131 μm/ms were generally faster than in vivo velocities, which ranged from 33 to 95 μm/ms (Armstrong-James et al., 1992; Petersen et al., 2003; Reyes-Puerta et al., 2016; Vilarchao et al., 2018), although 200 μm/ms has been reported (Lippert et al., 2007). Conduction velocities in BC slices tend to be greater: 74–473 μm/ms (Scheuer et al., 2023), 160 μm/ms (Haupt, 2000), and 130 μm/ms (Laaris and Keller, 2002). This difference is likely because naturalistic stimuli recruit cortical neurons more gradually (Ma and Patel, 2021), while electrical stimulation in slices induces abrupt, synchronous depolarization. Although natural sensory responses reflect system-level function and longer-range connections, electrical stimulation in vitro reflects the underlying intracortical unmyelinated axonal conduction velocities and synaptic delays of microcircuit components. Repetitive stimulation (100–500 Hz) in vivo leaves velocities unaffected at 113 μm/ms (Ferezou et al., 2006; Margalit and Slovin, 2018), consistent with indistinguishable velocities between successive pairs of pulses (Extended Data Fig. 7-1*D*). It is also of interest that the early phase of evoked traveling waves in the BC are similar to our velocities while the prestimulus and late-phase traveling waves are slower (Gonzales et al., 2025). Finally, we note that for saltatory barrel-to-barrel propagation, the mean distance of ∼180 µm (septa + and barrel width sum from Fig. S1) and mean latency of ∼6 ms (Fig. 8*A*) give a mean inter-barrel velocity of 30 μm/ms, which is at the very low end of the range noted above for in vivo velocity, as well as late-phase traveling waves (Gonzales et al., 2025). Lower velocities in vivo are consistent with the hypothesis that inter-barrel communication follows a circuitous route.

### Anisotropic inter-barrel circuitry

Our experiments probed for directional preferences in communication between barrels and revealed spatial biases embedded in BC circuitry. Morphological studies have suggested that anisotropic arborization of individual L2/3 and L4 pyramidal neurons may implement directional preferences locally between barrels (Narayanan et al., 2015). The intrinsic anisotropy reported here of latency and paired-pulse depression may also contribute to directional preferences. These spatial profiles of inter-barrel communication could play a role in processing sensory input and contribute to the differences in processing of fast foveal versus slow exploratory whisking.

Neighbor-barrel response amplitudes and half-widths were isotropic but latencies were not: caudal latencies were longest and rostral shortest. The absence of intracortical anisotropy in half-width and amplitude contrasts with the finding of whisking and whisker touch anisotropy in vivo (Vilarchao et al., 2018; Hubatz et al., 2020). Anisotropy in L4 of responses to whisker input in vivo could reflect both intracortical and subcortical processing, whereas our results reflect intracortical processing in isolation from corticothalamic projections. Temporal encoding within the cortex could interact in complex ways with rate-based coding from subcortical areas. Thus, disparities between anisotropies of an isolated circuit and anisotropies in vivo offer insight into the roles of the different components of the system*.*

The asymmetry observed in neighbor-barrel response latency in the present study parallels the lower density of barrel projections from L4 neurons to L2/3 caudal neighbors (Bernardo et al., 1990). The connections responsible for our caudal latencies may function in inter-barrel integration during foveal protraction, and the connections responsible for our rostral latencies may function in integration during foveal retraction (Table 2). This may be related to the protraction phase being 30% longer in duration than the retraction phase (Bermejo et al., 2002; Leiser and Moxon, 2007). The retraction direction may require shorter latencies to keep up with the shorter duration of this phase. We observed this difference only for directions aligned to the higher-frequency foveal pattern.

### Anisotropic short-term depression

Synaptic depression is known to vary within spatially defined subpopulations at the barrel and subbarrel scale (Argunşah et al., 2026). We found that short-term plasticity also exhibits a directional bias in inter-barrel circuits. We observed paired-pulse depression with a significant dependence on IPI and significant variations between directions. Protraction is primarily directed along the whisker row axis but also may decline in elevation near the rostral-most setpoint for whisking against a vertical pole (Petersen et al., 2020). Whisker contact with an object is communicated to caudal neighbor barrels ∼10 ms after the adjacent whisker contacts the same object (Vilarchao et al., 2018). Thus, sensing an object during foveal protraction may be accompanied by processing of successive contacts spreading to caudal neighbor barrels. Activation spreading from a stimulated barrel to a caudal neighbor may employ this circuitry. In contrast, exploratory protraction in free air produces an increase rather than decrease in whisker elevation (Knutsen et al., 2005), relating to inter-barrel communication spreading in the dorsal direction. Figure 9*E* illustrates these relationships and Table 2 summarizes them to provide a rationale for our groupings.

IPIs of 20–100 ms are relevant to repetitive thalamocortical input from a whisker. This input is unevenly distributed over the 10–50 Hz range and is sublinearly boosted during thalamic processing in the 5–25 Hz whisking range (Sosnik et al., 2001). Repetitive input to L4 propagates to neighboring barrels, and depression during inter-barrel communication processes this input. We observed that this communication is more strongly depressed in circuitry putatively related to exploratory protraction, matching the caudoventral multiwhisker deflection direction, which was previously observed to activate most strongly (Vilarchao et al., 2018). The relatively strong initial depolarization of circuitry related to this exploratory direction suggests that it reacts strongly to the initial contact, after which subsequent contacts and activity are filtered out.

### Conclusion

Genetically targeted voltage sensor imaging has revealed population-level responses in BC that illuminate the mechanisms of communication between barrels. Inter-barrel communication between L4 excitatory neurons employs layer-specific pathways. Our results suggest that a synaptic circuit enables activity initiated in L4 of one barrel to spread to L4 of neighboring barrels. While we propose L2/3 as a likely conduit, future experiments using optogenetics will be important to corroborate this circuit (e.g., by silencing L2/3) and determine which layers route inter-barrel traffic. By aligning barrels with whiskers, we were able to resolve subtle variations that depend on direction and stimulation IPI. The anisotropy in latency and synaptic depression seen here suggests that intracortical connections within BC have the potential to tune intracortical communication to the spatiotemporal structure of whisker input. Extending this line of investigation to other genetically defined cell types, to the single-cell level with sparse CreERT2-driven labeling (Feil et al., 2009; Shu and Jackson, 2024), to single-cell stimulation (Canales et al., 2022), and to optogenetic stimulation, has the potential to reveal the detailed circuitry underlying the inter-barrel signaling seen here and to identify the precise synapses, particularly in the L4→L2/3→L4 parts of the circuit. Furthermore, the strong inhibitory and disinhibitory actions of NBQX suggest that further imaging experiments with pharmacological manipulations have the potential to probe the detailed roles of specific circuit elements in intracortical communication.

## Synthesis

Reviewing Editor: Arianna Maffei, Stony Brook University Decisions are customarily a result of the Reviewing Editor and the peer reviewers coming together and discussing their recommendations until a consensus is reached. When revisions are invited, a fact-based synthesis statement explaining their decision and outlining what is needed to prepare a revision will be listed below. The following reviewer(s) agreed to reveal their identity: Krishna Jayant.

The study is overall considered very positively by both reviewers, who praised the importance of the question, the quality of the experimental design and analysis, and the relevance of the results to the field. There are concerns about some of the results presentations, which appear inconsistent with the description provided in the text, and with some of the conclusions, that appear overstated in view of the results reported in the manuscript. Some of the description of the findings appear not sufficiently precise to convey a clear description of the data analysis and findings. The relevance of a few of the measured parameters, including the latency of responses and half width of responses, as well as the meaning of direct and indirect activation of barrels is hard to follow. The strategies for normalization of data and presentation of heat maps are not clearly described. Finally, the discussion of the relevance of results obtained with reduced preparation for behavior is highly speculative and should be related more precisely to current literature on the topic.

The detailed comments by the reviewers follow below.

Reviewer #1

Main results and critiques:

1. L4principal▫ L2/3 ▫ L4Neighbor circuit anatomy

Key results demonstrate that activity initiated in the L4 of principal barrel spreads to the neighboring barrels via layer 2/3, thus forming L4principal▫ L2/3 ▫ L4Neighbor circuit. This finding is anatomically supported as the number of axonal projections from principal to neighboring L4 is outnumbered by principal to neighbor L2/3 projections. The stimulation paradigm is shown in Figure 2, and Figure 2G strongly corroborates the connectivity claim by showing that stimulation in L2/3 spreads more to neighboring barrels than stimulation in L4.

These results need to be corroborated by optogenetic controls. The exact circuit from L2/3 ▫ L4 remains unclear. How exactly is this synapse established?

2. Difference in half-width measures between L4 and L2/3 stim.

Certain details in Figure 2H are not discussed in the main text. The near doubling of the response half-width in neighbor versus home barrels is an interesting and unexplained finding. Whether this reflects polysynaptic recruitment, reduced inhibition in neighbor barrels, or differences in recruited neuronal populations is not addressed and warrants discussion.

How these results contrast with recent results on traveling waves across barrels should also be discussed in this context

3. Stimulation-evoked response latency

Latency from stimulation to half-maximum of rising phase was 1.9 ms for L4 home barrel responses to L4 stimulation, 3.0 ms for L2/3 Home Barrel, 3.9 ms for L2/3 neighbor, and 6.2 ms for L4 neighbor, which most strongly supports the circuit claim.

Once again, clear optogenetic controls are needed to support this circuit. To this reviewer, the inter-barrel spread to L4 is not clear.

4. Directional isotropy and anisotropy of neural activity properties.

Analyses of neural response properties as a function of the spatial organization of the barrels revealed isotropy in parameters such as response amplitude, half-width, and conduction velocity. However, anisotropy in response latency was observed in the rostral-caudal direction for neighboring barrels, where caudal neighbor barrels had higher integration latency than rostral barrels.

Once again, this reviewer would urge the authors to examine recent literature on traveling waves to support the measured latencies and velocities. Are these velocities consistent with unmyelinated axon conduction delays?

5. Short term plasticity

Paired-pulse depression experiments demonstrated strong directional anisotropy in short-term plasticity. Protraction-related directions (ventral and caudal) showed less depression and slower recovery in contrast to the strong depression related to retraction-associated directions (dorsal and rostral). The authors argue this preserves temporal fidelity during the information-rich protraction phase of whisking while strongly filtering repetitive input during retraction.

Exactly how? The result seems to be unclear. Perhaps a more detailed explanation will help this reviewer.

Further Major Comments:

1. The ecological validity of electrical stimulation should be considered while making behaviorally relevant claims. The authors note that stimulation induces large-scale synchronous depolarization, whereas real whisking activity produces sparse, temporally distributed firing. Therefore, it is difficult to assess how well the circuit dynamics revealed in this study will continue to operate under physiological conditions. A more explicit discussion of this gap will be beneficial for this paper. The latter part of the abstract, from "Such biases" onwards, needs to tone down the claim's behavioral relevance.

2. The authors acknowledge L5 contributions and the potential role of L6 corticothalamic neurons that receive input from L4. Immunohistochemical assays showing which cortical layers are preserved in the slice will help strengthen the claims.

3. Animals ranged from 4 to 13 weeks of age. This is a substantial cortical maturation window in mice. It is not stated whether age was included as a covariate in the mixed-effects linear regression model used to rule out pseudo-replication.

4. The Scnn1a tagging targets about 50% of layer 4 excitatory cells. This should be mentioned in the main text of the paper while drawing conclusions.

5. No single previous study has demonstrated the complete L4▫L2/3▫L4 inter-barrel relay as a functional circuit motif, ascending L4▫L2/3 circuit within a barrel and horizontal inter-barrel spread through L2/3 are anatomically well established by prior studies. Studies suggesting that L2/3 neurons closer to L4 have axonal projections to L4 need to be explicitly referenced or optogenetically verified. All these findings will further support the author's claims regarding the L4 ▫ L2/3 ▫ L4 mechanism. That said, anatomical connectivity following the L4home ▫ L2/3home ▫ L2/3neighbor ▫ L4neighbor path requires to be proved.

6. Recent studies using simultaneous 2-photon imaging and electrophysiology corroborate the circuit motif dynamics demonstrated here. Analyses of barrel cortex data suggest that a sparse network of strongly connected excitatory neurons in L2/3 supports sensory-evoked cortical traveling waves, thus showing that L2/3 activity can drive inter-barrel relay. The latencies (1.9-6.2ms) reported in this study recapitulate the early-onset traveling waves reported in the literature, which occur within 50ms of whisker touch in awake, behaving mice. However, literature also demonstrates that behaviorally relevant late traveling waves were modulated by stimulus value, task performance, and whisker motor feedback. These circuits are absent from the slice preparation by design; thus, the present study only better recapitulates the early feedforward substrate for inter-barrel propagation. This substantially limits the strength of the behavioral claims made in the study. Authors should discuss this limitation.

Reviewer #2

1

The authors stated: >> The present study focuses primarily on responses to direct activation of Scnn1A neurons by stimulation of L4.

"Direct stimulation" refers to the depolarization of neurons by the applied electric field, which alters the membrane potential independently of synaptic transmission. However, in the experiment shown in Figure 2, the authors cannot exclude a contribution from synaptically mediated responses within the home barrel, as electrical stimulation can also evoke neurotransmitter release from nearby neurons. Therefore, the statement that the "present study focuses primarily on responses to direct activation" is not fully supported, since both direct and synaptic components are likely to contribute to the observed signals.

2

The authors stated: >> This fit converged for 7 of 8 home barrels (mean R² = 0.93 {plus minus} 0.03) and 12 of 14 neighbor barrels (mean R2 = 0.88 {plus minus} 0.03).

The phrase "8 home barrels" is not sufficiently clear.

Did the authors want to say: "8 home barrels from 8 brain slices cut in both coronal and sagittal planes"?

3

The authors stated: >> The response amplitudes in the home (blue traces) and neighbor barrels (orange and pink traces) increased with stimulation current and saturated with strong stimulation.

However, this statement appears inconsistent with the data presented. In Fig. 3F (left panel), the response does not clearly reach a saturation, and therefore does not convincingly demonstrate saturation within the tested stimulation range.

4

In Fig. 4C, the optical signal amplitude appears to increase under the NBQX condition, as shown in the trace display. However, this seems inconsistent with the corresponding heat maps (peak ΔF/F maps). Specifically, during ACSF perfusion the home barrel contains multiple pixels with high amplitudes (red/yellow), whereas under NBQX these high-amplitude pixels are reduced or absent.

This raises a discrepancy: if both the heat maps and the traces are presented in ΔF/F units, it is unclear how the trace amplitude can be greater in the NBQX condition when the spatial maps (ACSF and NBQX)suggest reduced peak values after NBQX application. The authors should clarify whether the color scales are normalized independently for each condition, or whether differences in ROI averaging, scaling, or display normalization could account for this apparent inconsistency.

5

Figure 4E introduces substantial ambiguity due to several omissions in labeling and schematic clarity:

+++5-1. The figure does not indicate which structures correspond to the "home" versus "neighbor" barrels. Explicit labeling of these compartments is necessary for readers to correctly interpret the circuit.

+++5-2. The location of the stimulation site is not specified. It should be clearly marked which barrel receives electrical stimulation, as this is critical for understanding the directionality of the depicted signaling pathways.

+++5-3. The schematic omits representation of L4 excitatory neurons in both the home and neighbor barrels. Given that the study targets Scnn1a-expressing L4 neurons, both L4 compartments should include these cells, with their axonal projections appropriately illustrated toward L2/3 in both barrels. Consider green L4 cells with axons projecting into L2/3 boxes of both the Home and the Neighbor.

Addressing these issues would significantly improve the interpretability and accuracy of the figure.

6

The authors state: >> "Difference maps (denoted with 'Δ') display the change in peak ΔF/F resulting from NBQX addition for each pixel (white: no change; red: increase; blue: decrease)."

However, the example shown in Fig. 5A (bottom row) raises concerns about the consistency of this description. The difference map is defined as Δ = NBQX − ACSF. In the neighboring barrel, the observed blue perimeter is consistent with this definition, as synaptically mediated responses present in ACSF would be reduced under NBQX.

In the Difference map (Delta), at the stimulation site, we observe a red (positive) area indicating that signal in NBQX was greater than in the ACSF condition at this site. A quick examination of the Heat maps (Peak ΔF/F maps for ACSF &NBQX) shows that the Home barrel was significantly more depolarized in the ACSF than in the NBQX condition (Red amplitude in ACSF and Yellow amplitude in NBQX). Therefore, the subtraction procedure (Delta = NBQX minus ACSF) cannot produce the Difference map that was displayed in the figure.

7

The authors stated: >> By contrast, for L4 stimulation responses do not appear biphasic Fig. 4B), and in the home barrel (where amplitudes were not blocked by NBQX) a small reduction in half-width was seen (15.2 {plus minus} 2.3 to 11.7 {plus minus} 4.7 ms) that was not significant.

Please provide interpretation of this result. Why are L2/3 stimulation responses biphasic, while the L4 stimulation responses are not biphasic?

8

The authors stated: >> These qualitative boundary changes in velocity mark discrete transitions from direct activation of the home barrel to indirect activation of the neighboring barrel, reflecting parallel paths of inter-barrel communication, including mono- or poly-synaptic signaling through L2/3 (Fig. 7C).

This phrasing is difficult to interpret and would benefit from clarification. First, the term "direct activation" of the home barrel is ambiguous. Electrical stimulation is likely to evoke both direct depolarization and synaptically mediated responses, and it is not clear how the authors distinguish or isolate a purely "direct" component in this context.

Second, the meaning of "indirect activation" of the neighboring barrel is unclear. If this term is intended to describe activation via synaptic transmission, it would be more precise to state this explicitly: "activation via synaptic transmission".

Finally, the inclusion of "mono- or poly-synaptic signaling" adds complexity without clear justification, as the study does not distinguish between these possibilities. Referring more generally to "synaptic signaling" would improve clarity without sacrificing accuracy.

Overall, simplifying and defining these terms would make the interpretation more accessible and scientifically precise.

9

Figure 8 is difficult to interpret in its current form and would benefit from improved visualization. In particular, a three-dimensional schematic of the mouse head illustrating the relevant whisker movements would greatly aid comprehension. This could include: (1) rostro-dorsal exploratory whisking, corresponding to coronal slice analyses, and (2) foveal protraction, corresponding to sagittal slice analyses.

At present, it is challenging to conceptualize how the four directions of propagation (ventral, dorsal, rostral, caudal) relate to whisker movement and barrel organization. A clear mapping between slice axes, cortical directions, and whisker kinematics is needed to guide the reader.

Additionally, the authors state: "In vivo studies showed that deflection-evoked signals travel faster along rows than arcs." It is unclear which data points in Fig. 8 correspond to these directions (i.e., rows vs arcs), and how this comparison should be interpreted in the context of the present results. The figure and/or text should explicitly identify these relationships to avoid confusion, and enable meaningful comparison with prior in vivo findings.

10

The authors stated: >>This indicates that paired pulse depression is a consistently weaker, and signaling more persistent, for protraction-related directions of inter-barrel communication.

Which data points in Fig. 8 cover the "protraction-related directions of inter-barrel communication"?

11

The authors stated: >> During the protraction phase of free whisking this weaker paired-pulse depression in the caudal direction may preserve temporal fidelity for information moving along this specific direction.

Which data points in Fig. 8 cover the "protraction phase of free whisking"?

12

>> Fig. 9E clarifies a possible source of confusion caused by the alignment offset between whisker and barrel directions.

However, Fig. 9E introduces additional ambiguity rather than resolving it. The schematic presents three directions, whereas Fig. 8 analyzes four directions (ventral, dorsal, rostral, and caudal). This mismatch creates several inconsistencies. First, the number of directions differs between the two figures (three in Fig. 9E versus four in Fig. 8). Second, the "Ventral" direction discussed in Fig. 8 is not represented in Fig. 9E. Third, Fig. 9E appears to depict two distinct Rostral directions, whereas Fig. 8 includes only a single rostral category.

These discrepancies make it difficult to reconcile the conceptual framework across figures. The authors should revise the schematic to ensure a one-to-one correspondence between the directions illustrated in Fig. 9E and those analyzed in Fig. 8, and clearly define how each direction maps onto whisker kinematics and barrel cortex coordinates.

13

In FIG. 1. Caption, the authors wrote: >> B-C. Top: low-magnification fluorescence and gradient contrast images superimposed from R-C (B) and C-C (C) slices, with annotations for barrel, barrel relationship, and orientation.

However, the source of the fluorescence signal is not specified. The authors should clearly identify the labeling used in the image (e.g., hVOS expression in Scnn1a-expressing neurons).

14

The following sentence is unclear and appears to be corrupted, rendering it difficult to interpret.

We analyzed data from 11 male (21 slices, 48 barrels) and 11 female (23 slices,

56 barrels) mice aged 4-13 weeks aged 7.8 {plus minus} 1.7 weeks (2-3 barrels per slice, 1-4 slices per animal).

## Author Response

Dear Dr. Maffei Thank you for inviting us to submit a revised manuscript. We have addressed all of the reviewers' points, except for conducting optogenetic experiments, which was not feasible in the time allotted for resubmission. We acknowledge in the Discussion and Conclusion that optogenetics will be necessary to corroborate our conclusions. Broadly, our changes include toning down the claims about behavior, qualifying our hypothesis about the L4▫L2/3▫L4 relay, and addressing all the questions about the clarity of methods and results.

In the course of making revisions we re-examined the anatomy and realized we had overlooked the inversion of the row-to-barrel projection. We have corrected this oversight, making changes in the last two sections of Results and last two sections of Discussion. We also simplified the statistical analysis to make it clearer. New and heavily edited text is bold. Many small changes were made for clarity throughout the manuscript and these were not tracked to avoid clutter. We moved text from a Supplementary file to the main body of the manuscript per Journal style (highlighted in gray).

We thank the reviewers for their suggestions and feel that the revised paper is much improved. The reviews and our detailed responses follow.

Sincerely Meyer Jackson Synthesis Statement for Author (Required):

The study is overall considered very positively by both reviewers, who praised the importance of the question, the quality of the experimental design and analysis, and the relevance of the results to the field. There are concerns about some of the results presentations, which appear inconsistent with the description provided in the text, and with some of the conclusions, that appear overstated in view of the results reported in the manuscript. Some of the description of the findings appear not sufficiently precise to convey a clear description of the data analysis and findings. The relevance of a few of the measured parameters, including the latency of responses and half width of responses, as well as the meaning of direct and indirect activation of barrels is hard to follow. The strategies for normalization of data and presentation of heat maps are not clearly described. Finally, the discussion of the relevance of results obtained with reduced preparation for behavior is highly speculative and should be related more precisely to current literature on the topic.

Reviewer #1 Main results and critiques:

1. L4principal▫L2/3 ▫L4Neighbor circuit anatomy Key results demonstrate that activity initiated in the L4 of principal barrel spreads to the neighboring barrels via layer 2/3, thus forming L4principal▫L2/3▫L4Neighbor circuit. This finding is anatomically supported as the number of axonal projections from principal to neighboring L4 is outnumbered by principal to neighbor L2/3 projections. The stimulation paradigm is shown in Figure 2, and Figure 2G strongly corroborates the connectivity claim by showing that stimulation in L2/3 spreads more to neighboring barrels than stimulation in L4.

These results need to be corroborated by optogenetic controls. The exact circuit from L2/3▫L4 remains unclear. How exactly is this synapse established? Optogenetic experiments would provide definitive proof for our proposed circuit but this would require a major investment in technology development and require much more than 3 months. The Synthesis of Reviews did not mention optogenetics, presumably in recognition of the challenges of doing such experiments. We incorporated the reviewer's reservations into the Discussion along with a statement about how optogenetics is an important future direction that will provide critical corroboration (start of Discussion and Conclusion).

2. Difference in half-width measures between L4 and L2/3 stim.

Certain details in Figure 2H are not discussed in the main text. The near doubling of the response half-width in neighbor versus home barrels is an interesting and unexplained finding. Whether this reflects polysynaptic recruitment, reduced inhibition in neighbor barrels, or differences in recruited neuronal populations is not addressed and warrants discussion.

How these results contrast with recent results on traveling waves across barrels should also be discussed in this context We expanded the text regarding Fig. 2H and now discuss the doubling of the half-width more fully in terms of polysynaptic recruitment and differences in inhibition (Results: Barrel responses to stimulation of different layers, paragraph 2). We also added some explanation to our interpretation of NBQX halfwidths (Results: AMPA-receptor blockade identifies postsynaptic responses, final paragraph). Reviewer #2 (point 7) had similar questions about half-widths and these changes also address those concerns.

The reviewer asked for a discussion of traveling waves, both here and in a number of the points below. We agree that traveling waves are relevant and added a discussion to the Intracortical Velocity section of the Discussion, along with a comparison of their velocity with the velocities we reported. However, given the concerns expressed from the editor and reviewers about overstating the relevance of our reduced preparation to behavior, we limited the scope of this discussion because traveling waves are evoked by sensory input in behaving animals.

3. Stimulation-evoked response latency Latency from stimulation to half-maximum of rising phase was 1.9 ms for L4 home barrel responses to L4 stimulation, 3.0 ms for L2/3 Home Barrel, 3.9 ms for L2/3 neighbor, and 6.2 ms for L4 neighbor, which most strongly supports the circuit claim.

Once again, clear optogenetic controls are needed to support this circuit. To this reviewer, the inter-barrel spread to L4 is not clear.

We have incorporated this point about latencies in Results "Propagation through layers and barrels" last paragraph. We have attempted to make the possible mechanisms clearer. As noted above, optogenetic stimulation is out of the scope of this study and is mentioned in Discussion and Conclusion.

4. Directional isotropy and anisotropy of neural activity properties.

Analyses of neural response properties as a function of the spatial organization of the barrels revealed isotropy in parameters such as response amplitude, half-width, and conduction velocity. However, anisotropy in response latency was observed in the rostral-caudal direction for neighboring barrels, where caudal neighbor barrels had higher integration latency than rostral barrels.

Once again, this reviewer would urge the authors to examine recent literature on traveling waves to support the measured latencies and velocities. Are these velocities consistent with unmyelinated axon conduction delays? We incorporated a comparison of velocities of traveling waves into our survey of the literature on axonal conduction velocities in Discussion "Intracortical Velocity", where we noted that our velocities are typical of conduction in other cortical axons, as well as the early phase of traveling waves.

5. Short term plasticity Paired-pulse depression experiments demonstrated strong directional anisotropy in short-term plasticity. Protraction-related directions (ventral and caudal) showed less depression and slower recovery in contrast to the strong depression related to retraction-associated directions (dorsal and rostral). The authors argue this preserves temporal fidelity during the information-rich protraction phase of whisking while strongly filtering repetitive input during retraction.

Exactly how? The result seems to be unclear. Perhaps a more detailed explanation will help this reviewer.

We revised and clarified the presentation of Results "Barrel visualization and alignment," paragraph 2 and Results "Paired-pulse depression," paragraphs 4 and 5. We clarified the interpretation in Discussion "Anisotropic short-term depression" last paragraph, and Fig. 9E. We incorporated an additional related issue raised in Reviewer #2 comment 10.

Further Major Comments:

1. The ecological validity of electrical stimulation should be considered while making behaviorally relevant claims. The authors note that stimulation induces large-scale synchronous depolarization, whereas real whisking activity produces sparse, temporally distributed firing. Therefore, it is difficult to assess how well the circuit dynamics revealed in this study will continue to operate under physiological conditions. A more explicit discussion of this gap will be beneficial for this paper. The latter part of the abstract, from "Such biases" onwards, needs to tone down the claim's behavioral relevance.

We have extended our discussion of the difference between electrode-evoked and naturalistic stimuli, in the Discussion: "Intracortical Velocity" section. We also toned down claims of behavioral relevance in the abstract and throughout the paper.

2. The authors acknowledge L5 contributions and the potential role of L6 corticothalamic neurons that receive input from L4. Immunohistochemical assays showing which cortical layers are preserved in the slice will help strengthen the claims.

L6 corticothalamic neurons are particularly relevant due to their projections to L4 (Staiger &Petersen, 2021). All cortical layers are present in 200 µm thick BC slices. Both coronal and sagittal slices are cut perpendicular to the pial surface. We identified anatomical landmarks in our images at 2.5X magnification in fluorescent (Fig. 1) and bright field (not shown) images. This is now stated in Methods "Voltage imaging". In bright field images, barrel outlines are visible, and they are centered within the cortical columns extending to the subcortical boundary in each slice. This was cross-referenced with the 3D mouse barrel cortex atlas.

However, it is possible infragranular layers cross into barrels other than the ones we focused on in L4. This is especially an issue with lateral-aspect sagittal slices, which were excluded from the study for this reason. We added a comment to the "Mechanistic Insights" section of the Discussion that L6 circuitry would be a worthwhile future extension of this work. Immunohistochemical assays would be a useful addition to our referencing landmarks, atlases, and previous work.

3. Animals ranged from 4 to 13 weeks of age. This is a substantial cortical maturation window in mice. It is not stated whether age was included as a covariate in the mixed-effects linear regression model used to rule out pseudo-replication.

Animal was used as a random effect in the mixed-effects linear regression. It was found that animal-to-animal variation did not outweigh slice-to-slice variation. This indicated that animal age is not a source of variation and that using multiple slices from each animal does not result in pseudo-replication. We added this point to Methods "Analysis and Statistics" paragraph 3. Age and sex analyses are also given in Supplementary Results.

4. The Scnn1a tagging targets about 50% of layer 4 excitatory cells. This should be mentioned in the main text of the paper while drawing conclusions.

We added "Scnn1a-tg3-Cre targets about 50% of L4 excitatory neurons in L4 V1 (Wang et al., 2024)" to Results, paragraph 1.

5. No single previous study has demonstrated the complete L4▫L2/3▫L4 inter-barrel relay as a functional circuit motif, ascending L4▫L2/3 circuit within a barrel and horizontal inter-barrel spread through L2/3 are anatomically well established by prior studies. Studies suggesting that L2/3 neurons closer to L4 have axonal projections to L4 need to be explicitly referenced or optogenetically verified. All these findings will further support the author's claims regarding the L4▫L2/3▫L4 mechanism. That said, anatomical connectivity following the L4home▫L2/3home▫L2/3neighbor▫L4neighbor path requires to be proved.

We appreciate the Reviewer's recognition of the novelty of the L4▫L2/3▫L4 inter-barrel relay and now reference studies on lower L2/3 pyramidal neuron projections (Staiger &Petersen, 2021) in Results: "AMPA-receptor blockade identifies postsynaptic responses," paragraph 2 and Discussion: "Mechanistic insights into inter-barrel signaling", paragraph 2. While projections from upper L2/3 pyramidal neurons largely pass over L4 and L6 without synapsing, lower L2/3 pyramidal neurons have a smaller non-canonical feedback pathway to L4 that is rarely seen with paired recordings (Staiger &Petersen, 2021). We also acknowledge the importance of L6 CT▫L4 projections as pointed out in response to another point above (Results "AMPA receptor blockade" paragraph 2 and Discussion "Mechanistic insights into inter-barrel signaling" paragraph 2) 6. Recent studies using simultaneous 2-photon imaging and electrophysiology corroborate the circuit motif dynamics demonstrated here. Analyses of barrel cortex data suggest that a sparse network of strongly connected excitatory neurons in L2/3 supports sensory-evoked cortical traveling waves, thus showing that L2/3 activity can drive inter-barrel relay. The latencies (1.9-6.2ms) reported in this study recapitulate the early-onset traveling waves reported in the literature, which occur within 50ms of whisker touch in awake, behaving mice. However, literature also demonstrates that behaviorally relevant late traveling waves were modulated by stimulus value, task performance, and whisker motor feedback. These circuits are absent from the slice preparation by design; thus, the present study only better recapitulates the early feedforward substrate for inter-barrel propagation. This substantially limits the strength of the behavioral claims made in the study. Authors should discuss this limitation.

We have toned down the Abstract and Discussion and now emphasize that further work (optogenetics) is needed to establish these circuits and to connect the circuit mechanisms revealed in our study to behavior (Discussion and Conclusion). As noted above, the velocity of traveling waves was incorporated into Discussion "Intracortical Velocity".

Reviewer #2 1 The authors stated: >> The present study focuses primarily on responses to direct activation of Scnn1A neurons by stimulation of L4. "Direct stimulation" refers to the depolarization of neurons by the applied electric field, which alters the membrane potential independently of synaptic transmission. However, in the experiment shown in Figure 2, the authors cannot exclude a contribution from synaptically mediated responses within the home barrel, as electrical stimulation can also evoke neurotransmitter release from nearby neurons. Therefore, the statement that the "present study focuses primarily on responses to direct activation" is not fully supported, since both direct and synaptic components are likely to contribute to the observed signals.

We have added the reviewer's clarification of our stimulation method to Results "Barrel responses to stimulation of different layers" first paragraph.

2 The authors stated: >> This fit converged for 7 of 8 home barrels (mean R² = 0.93 {plus minus} 0.03) and 12 of 14 neighbor barrels (mean R2 = 0.88 {plus minus} 0.03).

The phrase "8 home barrels" is not sufficiently clear.

Did the authors want to say: "8 home barrels from 8 brain slices cut in both coronal and sagittal planes"? Yes we did. The reviewer's phrasing was incorporated into our text.

3 The authors stated: >> The response amplitudes in the home (blue traces) and neighbor barrels (orange and pink traces) increased with stimulation current and saturated with strong stimulation.

However, this statement appears inconsistent with the data presented. In Fig. 3F (left panel), the response does not clearly reach a saturation, and therefore does not convincingly demonstrate saturation within the tested stimulation range.

We changed the wording and now state that responses "approach a plateau". This reflects the fact that the increases diminish for larger stimulation currents. The logistic fits described in Results "Barrel responses to stimulation of different layers" yielded estimates of the saturating response. Our goal with this characterization in Fig. 3 was to ensure that we are studying the full extent of functional connections that we can access, and to implement a criterion for selection of stimulation current that is consistent between slices.

4 In Fig. 4C, the optical signal amplitude appears to increase under the NBQX condition, as shown in the trace display. However, this seems inconsistent with the corresponding heat maps (peak ΔF/F maps). Specifically, during ACSF perfusion the home barrel contains multiple pixels with high amplitudes (red/yellow), whereas under NBQX these high-amplitude pixels are reduced or absent.

This raises a discrepancy: if both the heat maps and the traces are presented in ΔF/F units, it is unclear how the trace amplitude can be greater in the NBQX condition when the spatial maps (ACSF and NBQX) suggest reduced peak values after NBQX application. The authors should clarify whether the color scales are normalized independently for each condition, or whether differences in ROI averaging, scaling, or display normalization could account for this apparent inconsistency.

For Fig. 4C, the reviewer is correct. While both colormaps were normalized to the same color scale, we had normalized values to the standard deviation of a pre-stimulation window of each trace, thus displaying signal-to-noise ratio (SNR) instead of ΔF/F. This difference indicates NBQX may increase variance. We have replaced the maps of SNR with maps of ΔF/F to remove this distraction.

5 Figure 4E introduces substantial ambiguity due to several omissions in labeling and schematic clarity: +++5-1. The figure does not indicate which structures correspond to the "home" versus "neighbor" barrels. Explicit labeling of these compartments is necessary for readers to correctly interpret the circuit.

We have added the requested labels to Fig. 4E. +++5-2. The location of the stimulation site is not specified. It should be clearly marked which barrel receives electrical stimulation, as this is critical for understanding the directionality of the depicted signaling pathways.

We updated Fig. 4E to illustrate stimulation site with an orange "spark" and use arrows to trace the sequence of excitation, creating one diagram for L2/3 stimulation and a separate diagram for L4 stimulation. +++5-3. The schematic omits representation of L4 excitatory neurons in both the home and neighbor barrels. Given that the study targets Scnn1a-expressing L4 neurons, both L4 compartments should include these cells, with their axonal projections appropriately illustrated toward L2/3 in both barrels. Consider green L4 cells with axons projecting into L2/3 boxes of both the Home and the Neighbor.

The left diagram of Fig. 4E shows L4 excitatory cell bodies in both barrels. We omitted L4 excitatory neurons in the neighbor barrel of the L4 stimulation diagram to reduce clutter, since neighbor barrel excitatory neurons do not have processes that are depolarized by L4 stimulation. We added text regarding their omission to the figure legend to clarify.

Addressing these issues would significantly improve the interpretability and accuracy of the figure.

We agree and hope our changes address these concerns.

6 The authors state: >> "Difference maps (denoted with 'Δ') display the change in peak ΔF/F resulting from NBQX addition for each pixel (white: no change; red: increase; blue: decrease)." However, the example shown in Fig. 5A (bottom row) raises concerns about the consistency of this description. The difference map is defined as Δ = NBQX − ACSF. In the neighboring barrel, the observed blue perimeter is consistent with this definition, as synaptically mediated responses present in ACSF would be reduced under NBQX.

In the Difference map (Delta), at the stimulation site, we observe a red (positive) area indicating that signal in NBQX was greater than in the ACSF condition at this site. A quick examination of the Heat maps (Peak ΔF/F maps for ACSF &NBQX) shows that the Home barrel was significantly more depolarized in the ACSF than in the NBQX condition (Red amplitude in ACSF and Yellow amplitude in NBQX). Therefore, the subtraction procedure (Delta = NBQX minus ACSF) cannot produce the Difference map that was displayed in the figure.

In the bottom row of Fig. 5A the peak in the ACSF map is red, while the peak is yellow in the NBQX map. The reviewer stated this looks like a reduction in response, which contradicts the difference map, where the red color indicates an increase. However, the peaks in the ACSF and NBQX maps are not at the same location. The peak in the NBQX map is a little to the left of the peak in the NBQX map. At the position of the yellow peak in the NBQX map, the ACSF map is green. So at this site the response in NBQX is indeed greater (because yellow>green).

To avoid this confusion we added the barrel and layer boundaries to the heat maps (they were already in the resting fluorescence image). These boundaries make the shift in peak easy to see. We also added a mention of the shift in peak to the text in Results "AMPA-receptor blockade identifies postsynaptic responses", paragraph 3).

7 The authors stated: >> By contrast, for L4 stimulation responses do not appear biphasic Fig. 4B), and in the home barrel (where amplitudes were not blocked by NBQX) a small reduction in half-width was seen (15.2 {plus minus} 2.3 to 11.7 {plus minus} 4.7 ms) that was not significant.

Please provide interpretation of this result. Why are L2/3 stimulation responses biphasic, while the L4 stimulation responses are not biphasic? There was a misunderstanding. The half-width values quoted by the reviewer were for neighbor barrels not home barrels. We saw that our text was not clear and this has been corrected.

Responses in L4 to L2/3 stimulation were often biphasic (Fig. 4D) because they were a mix of antidromic and synaptic responses. NBQX did not reduce their amplitude because of the dominating antidromic component. By contrast, NBQX reduced the amplitude of neighbor barrel responses to L4 stimulation by about 3-fold on average (Fig. 4D - right). The half-width of the neighbor barrel responses did not change significantly (the values cited above by the reviewer which are plotted in Fig. 4E - right). The lack of change noted by the reviewer suggests that the residual responses in NBQX arise predominantly from incompletely blocked synapses, and further suggests that antidromic responses do not make a detectable contribution to L4 neighbor-barrel responses elicited by L4 simulation. We addressed these points by revising Results "AMPA-receptor blockade identifies postsynaptic responses", final paragraph.

After addressing these concerns we noticed that the first sentence of the final paragraph of Discussion "Mechanistic insights into inter-barrel signaling" about NBQX and half-widths was closely related. Thanks to the deeper consideration of these issues prompted by the reviewer, we realized that this sentence was ill-considered and superfluous so we deleted it.

8 The authors stated: >> These qualitative boundary changes in velocity mark discrete transitions from direct activation of the home barrel to indirect activation of the neighboring barrel, reflecting parallel paths of inter-barrel communication, including mono- or poly-synaptic signaling through L2/3 (Fig. 7C).

This phrasing is difficult to interpret and would benefit from clarification. First, the term "direct activation" of the home barrel is ambiguous. Electrical stimulation is likely to evoke both direct depolarization and synaptically mediated responses, and it is not clear how the authors distinguish or isolate a purely "direct" component in this context.

We now state in our first use of stimulation that it depolarizes cell bodies, dendrites, and axons, and that responses represent direct, antidromic, and synaptic depolarizations of Scnn1A neurons. (Results "Barrel responses to stimulation of different layers" paragraph 1). The contributions are difficult to distinguish but a pharmacological test with NBQX helped resolve the contribution of excitatory synapses.

Second, the meaning of "indirect activation" of the neighboring barrel is unclear. If this term is intended to describe activation via synaptic transmission, it would be more precise to state this explicitly: "activation via synaptic transmission".

We removed "indirect" and now state that responses in the home barrel include direct depolarization, action potentials, and synaptic potentials, and that the neighboring barrel is activated through parallel paths of inter-barrel communication that depend on synaptic signaling through L2/3 (Results, "Intra- and inter-barrel velocity" paragraph 2).

Finally, the inclusion of "mono- or poly-synaptic signaling" adds complexity without clear justification, as the study does not distinguish between these possibilities. Referring more generally to "synaptic signaling" would improve clarity without sacrificing accuracy.

We agree that "indirect activation" and "mono- or poly-synaptic signaling" are unclear and we replaced them with the reviewer's suggested term "synaptic signaling" (Results "Intra- and inter-barrel velocity", paragraph 2).

Overall, simplifying and defining these terms would make the interpretation more accessible and scientifically precise.

9 Figure 8 is difficult to interpret in its current form and would benefit from improved visualization. In particular, a three-dimensional schematic of the mouse head illustrating the relevant whisker movements would greatly aid comprehension. This could include: (1) rostro-dorsal exploratory whisking, corresponding to coronal slice analyses, and (2) foveal protraction, corresponding to sagittal slice analyses.

At present, it is challenging to conceptualize how the four directions of propagation (ventral, dorsal, rostral, caudal) relate to whisker movement and barrel organization. A clear mapping between slice axes, cortical directions, and whisker kinematics is needed to guide the reader.

Additionally, the authors state: "In vivo studies showed that deflection-evoked signals travel faster along rows than arcs." It is unclear which data points in Fig. 8 correspond to these directions (i.e., rows vs arcs), and how this comparison should be interpreted in the context of the present results. The figure and/or text should explicitly identify these relationships to avoid confusion, and enable meaningful comparison with prior in vivo findings.

We struggled with how to conceptualize the four directions, and thank the reviewer for these suggestions. We created a 3D visualization and replaced Fig. 9E with a clearer and more illustrative schematic. The issues raised here are further discussed in the responses to the next two comments.

10 The authors stated: >>This indicates that paired pulse depression is a consistently weaker, and signaling more persistent, for protraction-related directions of inter-barrel communication.

Which data points in Fig. 8 cover the "protraction-related directions of inter-barrel communication"? The data points in groups Dorsal and Caudal correspond to protraction-related direction.

The clarification of the four directions suggested by the reviewer will help readers identify which data points correspond to which direction. This also enabled us to refer to these directions more clearly in the text (Results "Paired-pulse depression", paragraphs 4 and 5).

In our original analysis, we neglected to account for the inversion of barrel rows relative to the whisker rows (Wu et al., 2011). All other rotations and transformations were captured accurately by our reading of the 3D barrel cortex atlas. We re-did our analysis with the inversion correction. This also changed our behavioral interpretation, which we had intended to de-emphasize regardless (Last two sections of Discussion).

Dorsal slice directions, found in coronal slices, now correspond to exploratory retraction. Ventral slice directions, found in sagittal slices, correspond to exploratory protraction. Previously, we had mistakenly swapped these two directions by failing to account for row inversion.

Under our new assignment of protraction and retraction, the sum-contrast model confirmed but did not offer additional insight beyond our base analysis. We removed the sum-contrast analysis and instead focus on the analysis using ANOVA and Tukey's HSD.

11 The authors stated: >> During the protraction phase of free whisking this weaker paired-pulse depression in the caudal direction may preserve temporal fidelity for information moving along this specific direction.

Which data points in Fig. 8 cover the "protraction phase of free whisking"? The data points in groups Dorsal and Caudal correspond to protraction-related directions of inter-barrel communication.

Table 2 maps which directions in the data grouping correspond to which direction of stereotyped behavioral patterns. This is now stated in Results "Barrel visualization and alignment", paragraph 2, and Results "Paired-pulse depression", paragraphs 4 and 5, and illustrated in Fig. 9E.

12 >> Fig. 9E clarifies a possible source of confusion caused by the alignment offset between whisker and barrel directions.

However, Fig. 9E introduces additional ambiguity rather than resolving it. The schematic presents three directions, whereas Fig. 8 analyzes four directions (ventral, dorsal, rostral, and caudal). This mismatch creates several inconsistencies. First, the number of directions differs between the two figures (three in Fig. 9E versus four in Fig. 8). Second, the "Ventral" direction discussed in Fig. 8 is not represented in Fig. 9E. Third, Fig. 9E appears to depict two distinct Rostral directions, whereas Fig. 8 includes only a single rostral category.

These discrepancies make it difficult to reconcile the conceptual framework across figures. The authors should revise the schematic to ensure a one-to-one correspondence between the directions illustrated in Fig. 9E and those analyzed in Fig. 8, and clearly define how each direction maps onto whisker kinematics and barrel cortex coordinates.

The schematic has been revised to make Figs. 8 and 9 consistent. The original Fig. 9E included a distinction between rostral-aspect and caudal-aspect stereotyped whisking patterns which was not referenced in Fig. 8. This created the ambiguity that the Reviewer pointed out. To clear up this point, we replaced the schematic with a new one that does not include the caudal-aspect protraction. Instead, it has the retraction-oriented behavioral directions.

13 In FIG. 1. Caption, the authors wrote: >> B-C. Top: low-magnification fluorescence and gradient contrast images superimposed from R-C (B) and C-C (C) slices, with annotations for barrel, barrel relationship, and orientation.

However, the source of the fluorescence signal is not specified. The authors should clearly identify the labeling used in the image (e.g., hVOS expression in Scnn1a-expressing neurons).

The source of the fluorescence in the figure is now stated in the caption of Fig. 1 and Results "Barrel visualization and alignment", first paragraph.

14 The following sentence is unclear and appears to be corrupted, rendering it difficult to interpret.

We analyzed data from 11 male (21 slices, 48 barrels) and 11 female (23 slices, 56 barrels) mice aged 4-13 weeks aged 7.8 {plus minus} 1.7 weeks (2-3 barrels per slice, 1-4 slices per animal).

Our writing was over-compressed. Breaking it into two sentences made it clearer (Materials and Methods "Analysis and Statistics", paragraph 1).
